# Interaction-induced topological phase transition and Majorana edge states in low-dimensional orbital-selective Mott insulators

**DOI:** 10.1038/s41467-021-23261-2

**Published:** 2021-05-19

**Authors:** J. Herbrych, M. Środa, G. Alvarez, M. Mierzejewski, E. Dagotto

**Affiliations:** 1 Department of Theoretical Physics, Faculty of Fundamental Problems of Technology, Wrocław University of Science and Technology, Wrocław, Poland; 2 Computational Sciences and Engineering Division and Center for Nanophase Materials Sciences, Oak Ridge National Laboratory, Oak Ridge, TN USA; 3 Department of Physics and Astronomy, University of Tennessee, Knoxville, TN USA; 4 Materials Science and Technology Division, Oak Ridge National Laboratory, Oak Ridge, TN USA

**Keywords:** Magnetic properties and materials, Topological insulators

## Abstract

Topological phases of matter are among the most intriguing research directions in Condensed Matter Physics. It is known that superconductivity induced on a topological insulator’s surface can lead to exotic Majorana modes, the main ingredient of many proposed quantum computation schemes. In this context, the iron-based high critical temperature superconductors are a promising platform to host such an exotic phenomenon in real condensed-matter compounds. The Coulomb interaction is commonly believed to be vital for the magnetic and superconducting properties of these systems. This work bridges these two perspectives and shows that the Coulomb interaction can also drive a canonical superconductor with orbital degrees of freedom into the topological state. Namely, we show that above a critical value of the Hubbard interaction the system simultaneously develops spiral spin order, a highly unusual triplet amplitude in superconductivity, and, remarkably, Majorana fermions at the edges of the system.

## Abstract (web summary)

Superconductivity induced on a topological insulator’s surface may realize Majorana modes. Here, Herbrych et al. predict that a large Coulomb interaction helps develop a spiral spin order, which is a topological state at the edge of a canonical superconductor with orbital degrees of freedom.

## Introduction

Topologically protected Majorana fermions—the elusive particles which are their own antiparticles—are exciting because of their potential importance in fault-resistant quantum computation. From the experimental perspective, heterostructure-based setups were proposed as the main candidates to host the Majorana zero-energy modes (MZM). For example, the topologically protected gapless surface states of topological insulators can be promoted to MZM by the proximity-induced pairing caused by an underlying superconducting (SC) substrate^1^. However, the large spin–orbit coupling required to split the doubly degenerated bands due to the electronic spins, renders such a setup hard to engineer. Another group of proposals utilizes magnetic atoms (e.g., Gd, Cr, or Fe) arranged in a chain structure on a BCS superconductor^2–12^. These important efforts have shown that creating MZM in real condensed-matter compounds is challenging and only rare examples are currently available.

**Fig. 1:**
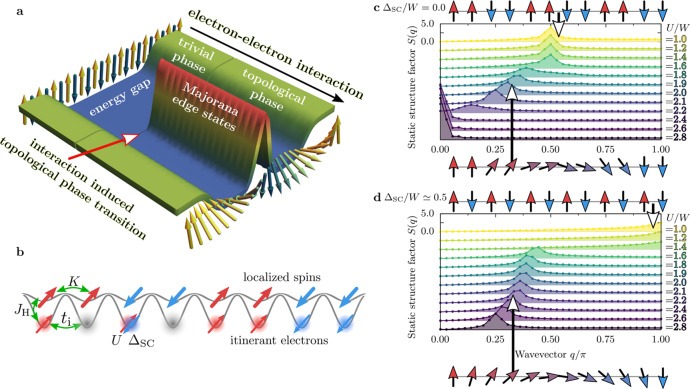
Orbital-selective Mott phase and Majoranas. **a** Sketch of the chain edge density-of-states as a function of the electron–electron Hubbard interaction strength. Magnetic orders (depicted by arrows) in the trivial and the topological superconducting (SC) phases are also presented. **b** Schematic representation of the generalized Kondo–Heisenberg model studied here, with localized and itinerant electrons and simultaneously active Hubbard *U* and superconducting Δ_SC_ couplings. **c** and **d** Interaction *U* dependence of the static structure factor *S*(*q*) for **c** Δ_SC_ = 0, **d** Δ_SC_/*W* ≃ 0.5 (calculated for *L* = 36 and $\overline{n}=0.5$).

Interestingly, a series of recent works have shown that doped high critical temperature iron-based superconductor Fe(Se,Te) can host MZM^13–17^. Although the electron–electron interaction is believed to be relevant for the pairing, its role in the stabilization of MZM is unknown. In fact, in most theoretical proposals to realize MZM, these zero-energy modes are a consequence of specific features in the non-interacting band structure, with the electron–electron interaction playing only a secondary role (and often even destabilizing the MZM)^18,19^. By contrast, here we will show that a SC system with orbital degrees of freedom can be driven into a topologically nontrivial phase hosting MZM via increasing Hubbard interactions; see illustrative sketch in Fig. 1a. We will focus on a generic model with coexisting wide and narrow energy bands, relevant to low-dimensional iron-based materials^20^. It was previously shown^21–23^ that the multi-orbital Hubbard model can accurately capture static and dynamical properties of iron selenides, especially the block-magnetic order^24^ of the 123 family AFe_2_X_3_ of iron-based ladders (with A alkali metals and X chalcogenides). For example, the three- and two-orbital Hubbard model on a one-dimensional (1D) lattice^23,25^ successfully reproduces the inelastic neutron scattering spin spectrum, with nontrivial optical and acoustic modes. The aforementioned models exhibit^21,26^ the orbital-selective Mott phase (OSMP), with coexistent Mott-localized electrons in one orbital and itinerant electrons in the remaining orbitals. The system is then in an exotic state with simultaneously metallic and insulating properties. Furthermore, the localized orbitals have vanishing charge fluctuations, simplifying the description^26^ into an OSMP effective model, i.e. the generalized Kondo–Heisenberg model (gKH)

1 $${H}_{{\rm{gKH}}}=	\; {t}_{{\rm{i}}}\mathop{\sum}\limits_{\ell ,\sigma }\left({c}_{\ell ,\sigma }^{\dagger }{c}_{\ell +1,\sigma }+{\rm{H.c.}}\right)+U\mathop{\sum}\limits_{\ell }{n}_{\ell ,\uparrow }{n}_{\ell ,\downarrow }\\ 	+\mu \mathop{\sum}\limits_{\ell ,\sigma }{n}_{\ell ,\sigma }-2{J}_{{\rm{H}}}\mathop{\sum}\limits_{\ell }{{\bf{S}}}_{\ell }\cdot {{\bf{s}}}_{\ell }+K\mathop{\sum}\limits_{\ell }{{\bf{S}}}_{\ell }\cdot {{\bf{S}}}_{\ell +1}.$$

The first three terms in the above Hamiltonian describe the itinerant electrons: ${c}_{\ell ,\sigma }^{\dagger }$ (${c}_{\ell ,\sigma }$) creates (destroys) an electron with spin projection *σ* = {↑, ↓} at site *ℓ* = {1, …, *L*}, *t*_i_ is their hopping amplitude, *U* is the repulsive Hubbard interaction, and *μ* = *ϵ*_F_ is the Fermi energy set by the density of itinerant electrons $\overline{n}={\sum }_{\ell }({n}_{\ell ,\uparrow }+{n}_{\ell ,\downarrow })/L$.

The double occupancy of the localized orbital can be eliminated by the Schrieffer–Wolff transformation and the remaining degrees of freedom, the localized spins **S**_*ℓ*_ in the above model, interact with one another via a Heisenberg term with spin-exchange $K=4{t}_{{\rm{l}}}^{2}/U$ [*t*_l_ is the hopping amplitude within the localized band]. Finally, *J*_H_ stands for the on-site interorbital Hund interaction, coupling the spins of the localized and itinerant electrons, **S**_*ℓ*_ and **s**_*ℓ*_, respectively. Figure 1b contains a sketch of the model. Here, we consider a 1D lattice and use *t*_i_ = 0.5 [eV] and *t*_l_ = 0.15 [eV], with kinetic energy bandwidth *W* = 2.1 [eV] as a unit of energy^27^. Furthermore, to reduce the number of parameters in the model, we set *J*_H_/*U* = 1/4, a value widely used when modeling iron superconductors. Systems with open boundary conditions are studied via the density-matrix renormalization group (DMRG) method (see the “Methods” section).

The key ingredient in systems expected to host the MZM^28^ is the presence of an SC gap, modeled typically by an *s*-wave pairing field. Such a term represents the proximity effect^29^ induced on the magnetic system by an external *s*-wave superconductor. However, it should be noted that the SC proximity effect has to be considered with utmost care. For example, recent experimental investigations^30^ showed that although the interface between Nb (BCS *s*-wave SC) and Bi_2_Se_3_ film (topological metal) leads to induced SC order, the same setup with (Bi_1−*x*_Sb_*x*_)_2_Se_3_ (another topological insulator) displays massive suppression of proximity pairing. On the other hand, in the class of systems studied here (low-dimensional OSMP iron-based materials), the pairing tendencies could arise from the intrinsic superconductivity of BaFe_2_S_3_ and BaFe_2_Se_3_ under pressure^31–33^ or doping^22,34^.

**Fig. 2:**
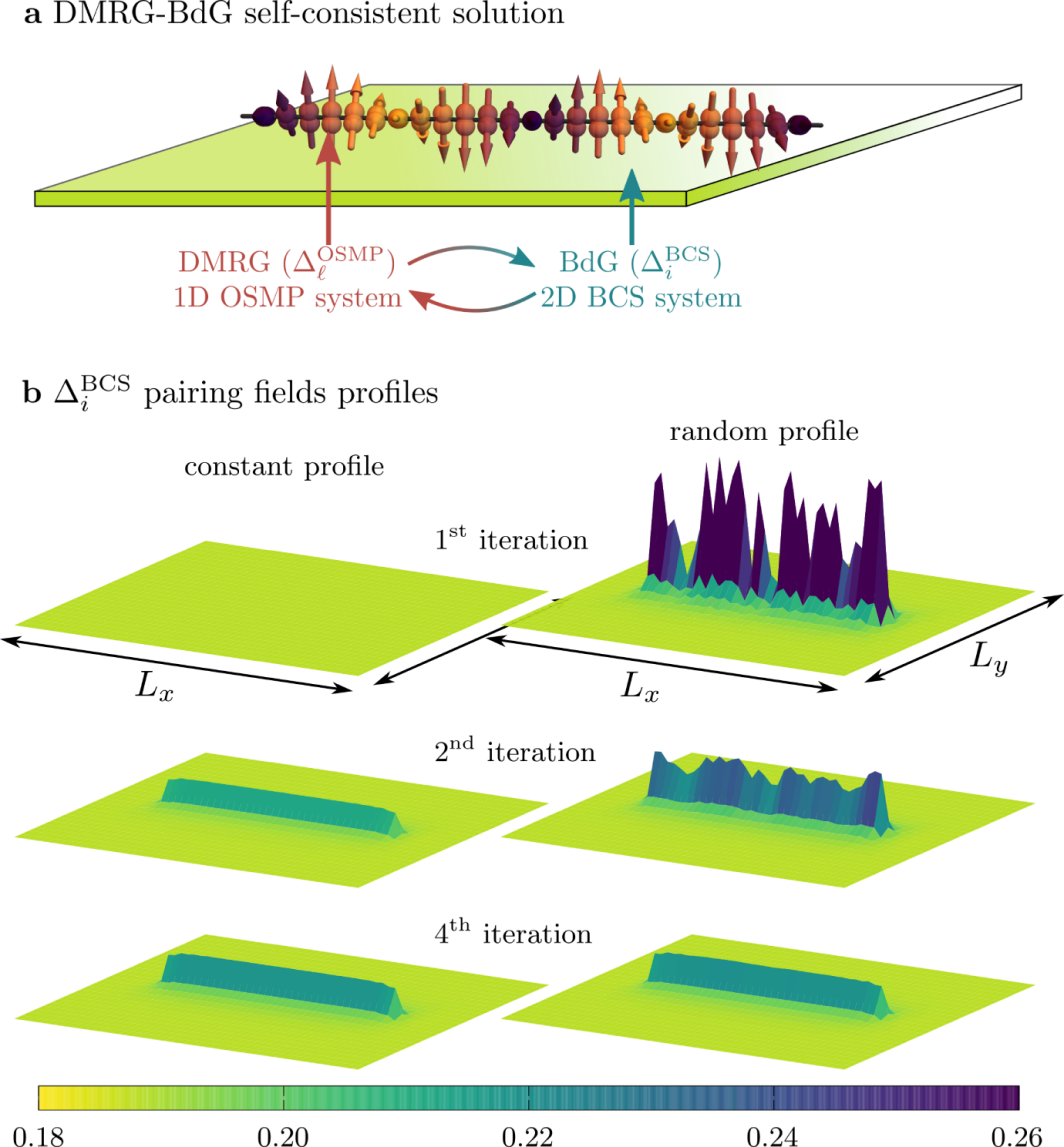
BdG self-consistent solution. **a** Sketch of the hybrid DMRG–BdG algorithm. The OSMP chain is placed in the middle of a 2D BCS superconductor and coupled to it via the pairing field amplitudes present in both systems. **b** Iteration dependence of the ${\Delta }_{i}^{{\rm{BCS}}}$ profiles for the case with the OSMP chain placed in the middle. The left (right) column depicts results initialized without (with random) pairing fields in the 1D OSMP system (calculated for *U*/*W* = 2). See the “Methods” sections for details.

In order to keep our discussion general, we will make minimal assumptions on the SC state, and consider only the simplest on-site pairing. The reader should consider it either as the intrinsic pairing tendencies of the iron-based SC material or as the pairing field induced by the proximity to an *s*-wave SC substrate, e.g., Pb or Nb. Independently of its origin, the SC in the 1D OSMP system studied here must be investigated beyond the 1D lattice since the quantum fluctuations would inevitably destroy any long-range order. Therefore, let us first consider the OSMP chain placed atop the center of a two-dimensional (2D) BCS superconductor (see Fig. 2a for a sketch) and the total system described by the Hamiltonian

2 $${H}_{{\rm{tot}}}={H}_{{\rm{gKH}}}+{H}_{{\rm{BCS}}}-V\mathop{\sum}\limits_{\langle \ell ,{\ell }^{\prime}\rangle }({c}_{\ell ,\uparrow }^{\dagger }{c}_{\ell ,\downarrow }^{\dagger }{a}_{{\ell }^{\prime},\downarrow }{a}_{{\ell }^{\prime},\uparrow }+{\rm{H.c.}}).$$

Here, ${\ell }^{\prime}$ represents the single site within the 2D BCS system *H*_BCS_ which is closest to the site *ℓ* in the OSMP chain, and ${a}_{i,\sigma },{a}_{i,\sigma }^{\dagger }$ stand for fermionic operators within the BCS superconductor (see the “Methods” section). The interaction between the subsystems [last term in Eq. (2)] is studied within the BCS-like decoupling scheme, where we introduce the pairing amplitudes ${\Delta }_{{\ell }^{\prime}}^{{\rm{BCS}}}=\langle {a}_{{\ell }^{\prime},\downarrow }{a}_{{\ell }^{\prime},\uparrow }\rangle$ and ${\Delta }_{\ell }^{{\rm{OSMP}}}=\langle {c}_{\ell ,\downarrow }{c}_{\ell ,\uparrow }\rangle$ for the BCS superconductor and the OSMP chain, respectively. In order to fully take into account the many-body nature of the OSMP system, we have developed a hybrid algorithm, the details of which are given in the “Methods” section. In summary: we iteratively solve the OSMP chain and the BCS system by means of the DMRG and the Bogoliubov–de Gennes (BdG) equations, respectively. This back-and-forth computational setup is costly but important to gain confidence in our result.

We monitor the landscapes of pairing fields in both systems and exemplary results are presented in Fig. 2b (for more results see Supplementary Note 1). Initially, only the BCS system has finite, spatially uniform, pairing amplitudes ${\Delta }_{{\ell }^{\prime}}^{{\rm{BCS}}}$ (left column in Fig. 2b), which are then used in the DMRG procedure applied to the OSMP Hamiltonian

3 $$H={H}_{{\rm{gKH}}}+\mathop{\sum}\limits_{\ell }{\Delta }_{\ell }\left({c}_{\ell ,\uparrow }^{\dagger }{c}_{\ell ,\downarrow }^{\dagger }+{\rm{H.c.}}\right),$$

where ${\Delta }_{\ell }=-V{\Delta }_{{\ell }^{\prime}}^{{\rm{BCS}}}$. Next, the ${\Delta }_{\ell }^{{\rm{OSMP}}}$ set is calculated from DMRG and returned to the BdG equations relevant for the BCS system. The procedure is repeated until convergence is established. The results presented in Fig. 2b show that already after ~4 iterations the landscape of ${\Delta }_{{\ell }^{\prime}}^{{\rm{BCS}}}$ stabilizes to an interaction *U*-dependent value. We found that the resulting amplitude ${\Delta }_{\ell }=-V{\Delta }_{{\ell }^{\prime}}^{{\rm{BCS}}}$ is almost uniform within the OSMP chain. Furthermore, we have also confirmed that using extended *s*-wave pairing (creating pairs on nearest-neighbor sites) does not influence our conclusions. Therefore, in the remainder of the paper, we use spatially uniform Δ_*ℓ*_ = Δ_SC_ in Eq. (3). Also, in order to emphasize the role of interaction, in the main text, we fix the pairing field to Δ_SC_/*W* ≃ 0.5. The detailed Δ_SC_-dependence of our findings is discussed in Supplementary Note 1.

## Results

### Magnetism of OSMP

Previous work has shown that the OSMP (with Δ_SC_ = 0) has a rich magnetic phase diagram^26^. (i) At small *U* the system is paramagnetic. (ii) At $\overline{n}=1$ and $\overline{n}=0$ standard antiferromagnetic (AFM) order develops, ↑↓↑↓, with total on-site magnetic moment 〈**S**^**2**^〉 = *S*(*S* + 1) = 2 and 3/4, respectively. (iii) For $0\;<\;\overline{n}\;<\;1$ and *U* ≫ *W* the system is a ferromagnet (FM) ↑↑↑↑. Interestingly, in the always challenging intermediate interaction regime $U \sim {\mathcal{O}}(W)$ the AFM- and FM-tendencies (arising from superexchange and double-exchange, respectively) compete and drive the system towards novel magnetic phases unique to multi-orbital systems. (iv) For *U* ~ *W*, the system develops a so-called block-magnetic order, consisting of FM blocks that are AFM coupled, e.g. ↑↑↓↓, as sketched in Fig. 1c. The block size appears controlled by the Fermi vector *k*_F_, i.e., the propagation wavevector of the block-magnetism is given by ${q}_{\max }=2{k}_{{\rm{F}}}$ (with $2{k}_{{\rm{F}}}=\pi \overline{n}$ for the chain lattice geometry). In this work, we choose $\overline{n}=0.5$ (adjusted via the chemical potential *μ*), as the relevant density for BaFe_2_Se_3 _*π*/2-block magnetic order^24^. Then, the latter order can be identified via the peak position of the static structure factor *S*(*q*) = 〈**T**_−*q*_ ⋅ **T**_*q*_〉 at ${q}_{\max }=\pi /2$ or via a finite dimer order parameter *D*_*π*/2_ = ∑_*ℓ*_(−1)^*ℓ*^〈**T**_*ℓ*_ ⋅ **T**_*ℓ*+1_〉/*L*, where we introduced the Fourier transform ${{\bf{T}}}_{q}={\sum }_{\ell }\exp (iq\ell )\ {{\bf{T}}}_{\ell }/\sqrt{L}$ of the total spin operator **T**_*ℓ*_ = **S**_*ℓ*_ + **s**_*ℓ*_. In Fig. 1c *S*(*q*) is shown at moderate interaction: at *U*/*W* < 1.6 it displays a maximum at ${q}_{\max }=\pi /2$, consistent with ↑↑↓↓ the order.

Remarkably, it has been shown recently^27^ that there exists an additional unexpected phase in between the block- and FM-ordering. Namely, upon increasing the interaction (1.6 < *U*/*W* < 2.4), the maximum of *S*(*q*) in Fig. 1c shifts towards incommensurate wavevectors (while for *U*/*W* > 2.4 the system is a ferromagnet). This incommensurate region reflects a novel magnetic spiral where the magnetic islands maintain their ferromagnetic character (with *D*_*π*/2_ ≠ 0) but start to rigidly rotate, forming a so-called block-spiral (see sketch Fig. 1c). The latter can be identified by a large value^27^ of the long-range chirality correlation function 〈***κ***_*ℓ*_ ⋅ ***κ***_*m*_〉 where ***κ***_*ℓ*_ = **T**_*ℓ*_ × **T**_*ℓ*+*N*_ and *N* is the block size. It is important to note that the spiral magnetic order appears without any direct frustration in the Hamiltonian (1), but rather is a consequence of hidden frustration caused by competing energy scales in the OSMP regime. Finally, it should be noted that the block-spiral OSMP state is not limited to 1D chains. In Supplementary Note 2, we show similar investigations for the ladder geometry and find rigidly rotating 2 × 2 FM islands. These results are consistent with recent nuclear magnetic resonance measurements on the CsFe_2_Se_3_ ladder compound which reported the system’s incommensurate ordering^35^.

Interestingly, an interaction-induced spiral order is also present when SC pairing is included in the model, as evident from Fig. 1d. However, the spiral mutates from block- to canonical-type with *D*_*π*/2_ = 0 (see the sketch in Fig. 1d), indicating unusual back-and-forth feedback between magnetism and superconductivity. As discussed below, the pairing optimizes the spiral profile to properly create the Majoranas. The competition between many energy scales (Hubbard interaction, Hund exchange, and SC pairing) leads to novel phenomena: an interaction-induced topological phase transition into a many-body state with MZM, unconventional SC, and canonical spiral.

### Majorana fermions

**Fig. 3:**
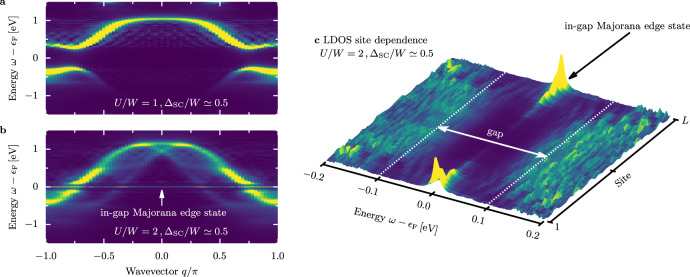
Spectral functions. Effect of the finite pairing field Δ_SC_/*W* ≃ 0.5 on the single-particle spectral function *A*(*q*, *ω*) for **a** *U*/*W* = 1 and **b** *U*/*W* = 2 calculated for *L* = 36, $\overline{n}=0.5$, and *δω* = 0.02 [eV]. Majorana zero-energy modes are indicated in **b**. **c** Local density-of-states (LDOS) in the in-gap frequency region (*δω* = 0.002 [eV]) vs. chain site index. The sharp LDOS peaks at the edges represent Majorana edge states, while the bulk of the system exhibits gapped behavior.

Figure 3 shows the effect of Δ_SC_/*W* ≃ 0.5 on the single-particle spectral function *A*(*q*, *ω*) (see the “Methods” section) for the two crucial phases in our study, the block-collinear and block-spiral magnetic orders (*U*/*W* = 1 and *U*/*W* = 2, respectively). As expected, in both cases, a finite SC gap opens at the Fermi level *ϵ*_F_ (~0.5 [eV] for *U*/*W* = 1 and ~0.1 [eV] for *U*/*W* = 2). Remarkably, in the block-spiral phase, an additional prominent feature appears: a sharply localized mode inside the gap at *ϵ*_F_, displayed in Fig. 3b. Such an in-gap mode is a characteristic feature of a topological state, namely the bulk of the system is gapped, while the edge of the system contains the in-gap modes. To confirm this picture, in Fig. 3c, we present a high-resolution frequency data of the real-space local density-of-states (LDOS; see the “Methods” section) near the Fermi energy *ϵ*_F_. As expected, for the topologically nontrivial phase, the zero energy modes are indeed confined to the system’s edges. It is important to note that this phenomenon is absent for weaker interaction *U*/*W* = 1. Furthermore, one cannot deduce this behavior from the *U* → *∞* or *J*_H_ → *∞* limits, where the system has predominantly collinear AFM or FM ordering, leading again to a trivial SC behavior. However, as shown below, at moderate *U* the competing energy scales present in the OSMP lead to the topological phase transition controlled by the electron–electron interaction.

**Fig. 4:**
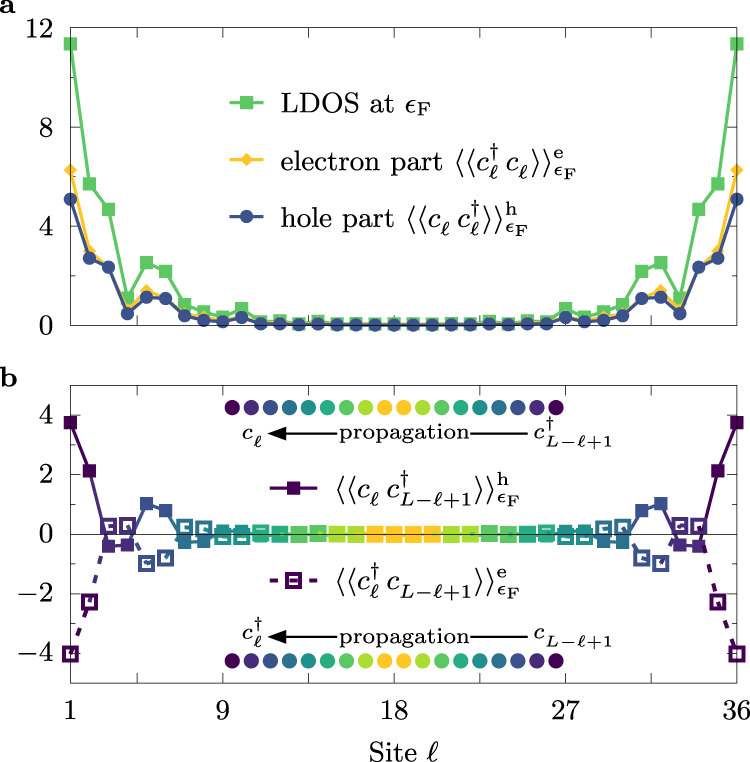
Correlation functions of Majorana fermions. **a** Site *ℓ* dependence of the local density-of-states (LDOS) at the Fermi level (*ω* = *ϵ*_F_) together with its hole ${\langle \langle {c}_{\ell }\ {c}_{\ell }^{\dagger }\rangle \rangle }_{{\epsilon }_{{\rm{F}}}}^{{\rm{h}}}$ and electron ${\langle \langle {c}_{\ell }^{\dagger }\ {c}_{\ell }\rangle \rangle }_{{\epsilon }_{{\rm{F}}}}^{{\rm{e}}}$ contributions. **b** Site dependence of the centrosymmetric spectral function ${\langle \langle {c}_{\ell }\ {c}_{L-\ell +1}^{\dagger }\rangle \rangle }_{{\epsilon }_{{\rm{F}}}}^{{\rm{h}}}$ and ${\langle \langle {c}_{\ell }^{\dagger }\ {c}_{L-\ell +1}\rangle \rangle }_{{\epsilon }_{{\rm{F}}}}^{{\rm{e}}}$. Sketches represent the calculated process: the probability of creating the electron on one end of the system (site *ℓ*) and a hole at the opposite end (site *L* − *ℓ* + 1), or vice-versa, at given energy *ω*. The pairs of sites where the spectral function is evaluated are represented by the same colors. All results were calculated for *L* = 36, *U*/*W* = 2, Δ_SC_/*W* ≃ 0.5, and $\overline{n}=0.5$.
(ii) The total spectral weight present in the localized modes can be rigorously derived from the assumption of the MZM’s existence (see the “Methods” section), and it should be equal to 0.5. Integrating our DMRG results in Fig. 3c over a narrow energy window and adding over the first few edge sites gives ≃ 0.47, very close to the analytical prediction. Note that the Majoranas are not strictly localized at one edge site *ℓ* ∈ {1, *L*}, as evident from Fig. 4a. Instead, the MZM is exponentially decaying over a few sites (see Fig. 5c), and we must add the spectral weight accordingly (separately for the left and right edges).

Let us now identify the induced topological state. The size dependence of the LDOS presented in Fig. 3c reveals zero-energy edge modes, namely peaks at frequency *ω* ≃ *ϵ*_F_ localized at the edges of the chain with open ends. While such modes are a characteristic property of the MZM, finding peaks in the LDOS alone is insufficient information for unambiguous identification. To demonstrate that the gKH model with superconductivity indeed hosts Majorana modes, we have numerically checked three distinct features of the MZM:

(i) Since the Majorana particles are their own antiparticles, the spectral weight of the localized modes should be built on an equal footing from the electron and hole components. Figure 4a shows that this is indeed the case.

**Fig. 5:**
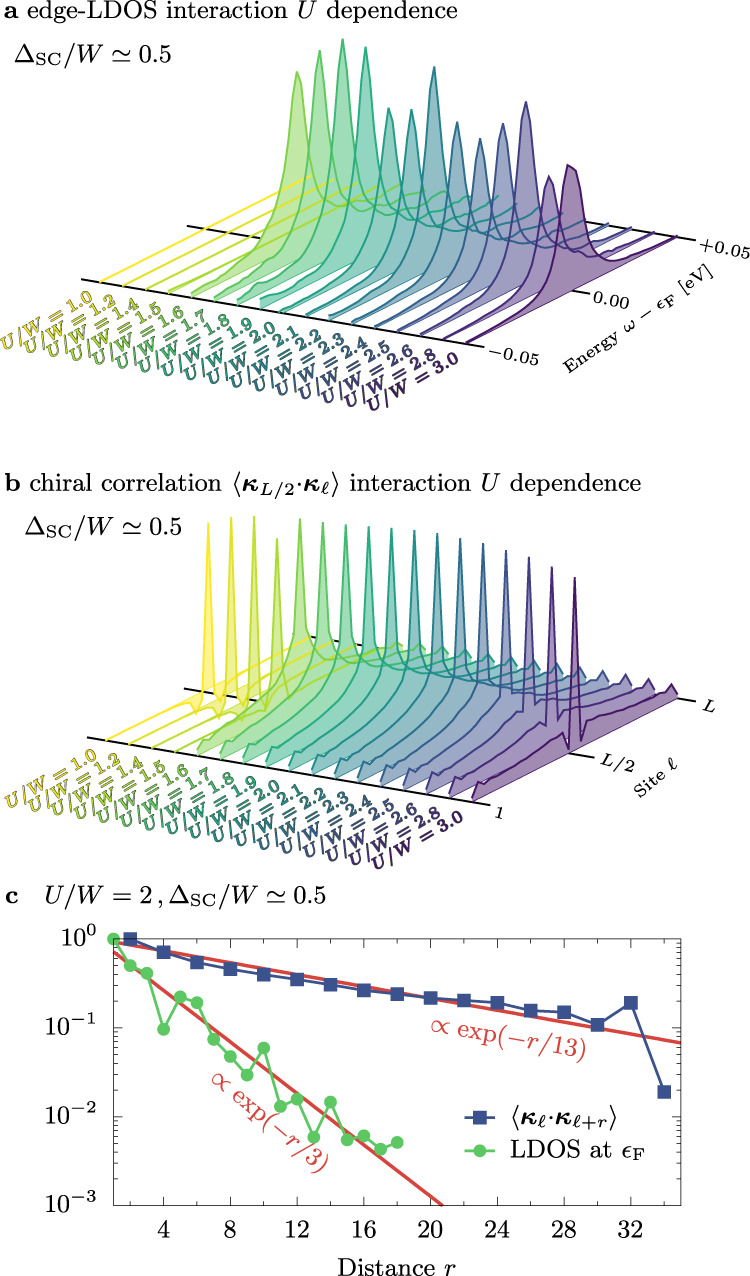
Interaction dependence of the MZM. Dependence on the Hubbard interaction *U* of **a** the edge-LDOS at site *ℓ* = 1 (near the Fermi level *ϵ*_F_) and **b** the chirality correlation function 〈***κ***_*L*/2_ ⋅ ***κ***_*ℓ*_〉. All results calculated for Δ_SC_/*W* ≃ 0.5, $\overline{n}=0.5$, *L* = 36. **c** Spatial decay of the local density-of-states at the Fermi level (LDOS at *ϵ*_F_) and the chirality correlation function 〈***κ***_*ℓ*_ ⋅ ***κ***_*ℓ*+*r*_〉 for *U*/*W* = 2. Red solid lines indicate exponential decay $\exp (-r/{l}_{\alpha })$ with *l*_MZM_ = 3 and *l*_s_ = 15, for the MZM and the spiral order, respectively.
(iii) The MZM located at the opposite edges of the system form one fermionic state, namely the edge MZM is correlated with one another over large distances. To show such behavior, consider the hole- and electron-like centrosymmetric spectral functions, ${\langle \langle {c}_{\ell }\ {c}_{L-\ell +1}^{\dagger }\rangle \rangle }_{\omega }^{{\rm{h}}}$ and ${\langle \langle {c}_{\ell }^{\dagger }\ {c}_{L-\ell +1}\rangle \rangle }_{\omega }^{{\rm{e}}}$, respectively. These functions represent the probability amplitude of creating an electron on one end and a hole at the opposite end (or vice-versa) at a given energy *ω* (see the “Methods” section for detailed definitions and Supplementary Note 3 for further discussion). Figure 4b shows ${\langle \langle {c}_{\ell }\ {c}_{L-\ell +1}^{\dagger }\rangle \rangle }_{\omega }^{{\rm{h}}}$ and ${\langle \langle {c}_{\ell }^{\dagger }\ {c}_{L-\ell +1}\rangle \rangle }_{\omega }^{{\rm{e}}}$ at the Fermi level *ω* = *ϵ*_F_, namely in the region where the MZM should be present. As expected, the bulk of the system behaves fundamentally different from the edges. In the former, crudely when *L*/2 ≲ *ℓ* ≲ 3*L*/4, the aforementioned spectral functions vanish reflecting the gapped (bulk) spectrum with lack of states at the Fermi level. However, at the boundaries (*ℓ* ≪ *L*/2 and *ℓ* ≫ *L*/2) the values of the centrosymmetric spectral functions are large, with a maximum at the edges *ℓ* ∈ {1, *L*}. The long-range (across the system) correlations of the edge states strongly support their topological nature.

Finally, let us discuss the physical mechanism causing the onset of MZM. In Fig. 5a we present the Hubbard *U* interaction dependence of the edge-LDOS (*ℓ* = 1) in the vicinity of the Fermi level, *ω* ~ *ϵ*_F_. It is evident from the presented results that the edge-LDOS acquires a finite value quite abruptly for *U* > *U*_c_ ≃ 1.5. To further clarify this matter, let us return to the magnetic states in the OSMP regime. Figure 5b shows the chirality correlation function 〈***κ***_*L*/2_ ⋅ ***κ***_*ℓ*_〉 (with ***κ***_*ℓ*_ = **T**_*ℓ*_ × **T**_*ℓ*+1_) for increasing value of the Hubbard *U* strength. We observe a sudden appearance of the chirality correlation exactly at *U*_c_, a behavior similar to that of the edge LDOS. Interestingly, in the system without the pairing field, Δ_SC_ = 0, at a similar value of *U* ≃ 1.6 the system enters the block-spiral phase with rigidly rotating FM islands. However, in our setup, the tendencies of OSMP to create magnetic blocks^26^ are highly suppressed by empty and doubly occupied sites favored by the finite pairing field Δ_SC_. As a consequence, the block-spiral order is reshaped to a canonical type of spiral without dimers *D*_*π*/2_ = 0. This behavior is similar to the MZM observed when combining *s*-wave SC with a classical magnetic moment heterostructure^2,4–6^. In the latter, the Ruderman–Kittel–Kasuya–Yosida (RKKY) mechanism stabilizes a classical long-range spiral with 2*k*_F_ pitch (where ${k}_{{\rm{F}}}\propto \overline{n}$ is the Fermi wavevector). Within the OSMP, however, the pitch is, on the other hand, controlled by the interaction *U* (at fixed $\overline{n}$), as evident from the results presented in Fig. 1b, c.

Furthermore, analysis of the chirality correlation function 〈***κ***_*ℓ*_ ⋅ ***κ***_*ℓ*+*r*_〉 indicates that the spiral order decays with the distance *r* (see Fig. 5c), as expected in a 1D quantum system. Note, however, that the MZM decay length scale, *l*_MZM_, and that of the spiral, *l*_s_, differ substantially. The Majoranas are predominantly localized at the system edges, thus yielding a short localization length *l*_MZM_ ≃ 3. The spiral, although still decaying exponentially, has a robust correlation length *l*_s_ ≃ 13, of the same magnitude as the Δ_SC_ = 0 result^27^. Then, for large but finite chains the overlap between the edge modes is negligible while the magnetic correlations on the distance *L* are still large enough to promote triplet pairing and the Majorana modes. In addition, we have observed that smaller values of Δ_SC_ than considered here also produce the MZM. However, since the Majoranas have an edge localization length inversely proportional to Δ_SC_, reducing the latter leads to overlaps between the left and right Majorana states in our finite systems^28,36^, thus distorting the physics we study. After exploration, Δ_SC_/*W* ≃ 0.5 was considered an appropriate compromise to address qualitatively the effects of our focus given our practical technical constraints within DMRG (see Supplementary Note 4 for details).

Conceptually, it is important to note that the interaction-induced spiral at *U*/*W* = 2 is not merely frozen when Δ_SC_ increases. Specifically, the characteristics^27^ of the chirality correlation function 〈***κ***_*i*_ ⋅ ***κ***_*j*_〉 qualitatively differ between the trivial (Δ_SC_ = 0) and topological phases (Δ_SC_ ≠ 0): increasing Δ_SC_ suppresses the dimer order and leads to a transformation from block spiral to a standard canonical spiral with *D*_*π*/2_ = 0 in the topologically nontrivial phase. As a consequence, the proximity to a superconductor influences on the magnetic order to optimize the spin pattern needed for MZM. Surprisingly, Δ_SC_ influences on the collinear spin order as well. In fact, at *U*/*W* = 1, before spirals are induced, the proximity to superconductivity changes the block spin order into a more canonical staggered spin order to optimize the energy (see Fig. 3b). This is a remarkable, and unexpected, back-and-forth positive feedback between degrees of freedom that eventually causes the stabilization of the MZM.

## Discussion

**Fig. 6:**
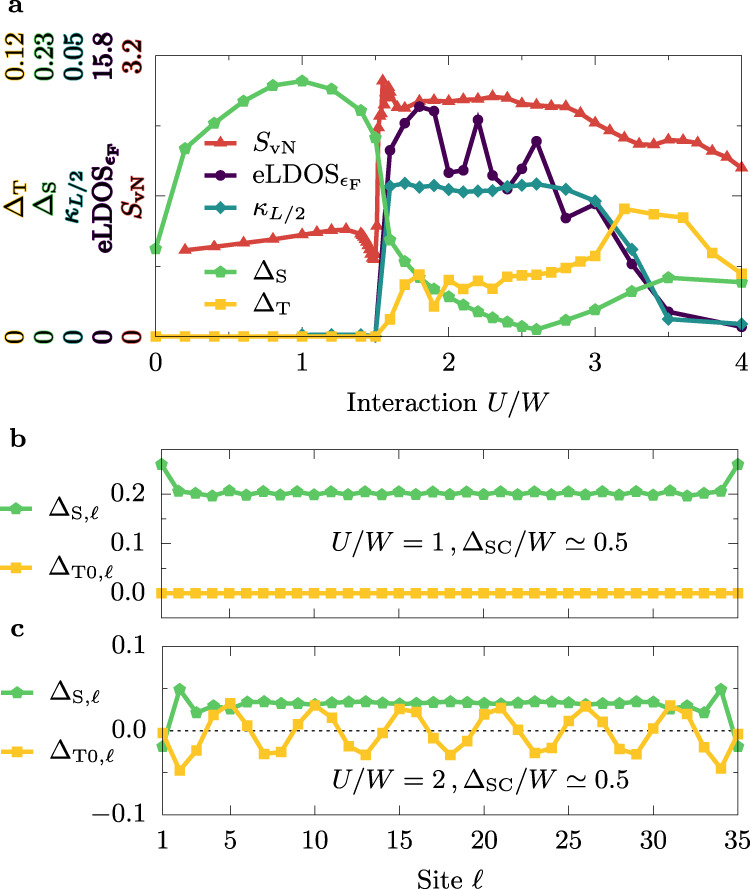
Phase diagram. **a** Hubbard interaction *U* dependence of the (i) von Neumann entanglement entropy *S*_vN_, (ii) edge local density-of-states at the Fermi level (eLDOS${}_{{\epsilon }_{{\rm{F}}}}$), (iii) the value of chirality correlation function at distance *L*/2 (i.e. 〈***κ***_*L*/4_ ⋅ ***κ***_3*L*/4_〉), as well as (iv) nonlocal singlet Δ_S_ and triplet Δ_T_ pairing amplitudes. See text for details. All results were calculated for *L* = 36, Δ_SC_/*W* ≃ 0.5, and $\overline{n}=0.5$. **b**, **c** Spatial dependence of the singlet and triplet SC amplitudes, Δ_S,*ℓ*_ and Δ_T0,*ℓ*_, respectively (see the “Methods” section for details), with **b** the trivial phase (*U*/*W* = 1, Δ_SC_/*W* ≃ 0.5) and **c** the topological phase (*U*/*W* = 2, Δ_SC_/*W* ≃ 0.5). In the latter, the oscillations of the triplet component are related to the pitch of the underlying spiral magnetic order.

Our main findings are summarized in Fig. 6: upon increasing the strength of the Hubbard interaction *U* within the OSMP with added SC pairing field, the system undergoes a topological phase transition. The latter can be detected as the appearance of edge modes which are mutually correlated in a finite system. This in turn leads to, e.g., the sudden increase of the entanglement, as measured by the von Neumann entanglement entropy *S*_vN_ (see the “Methods” section). The transition is driven by the change in the magnetic properties of the system, namely by inducing a finite chirality visible in the correlation function 〈***κ***_*ℓ*_ ⋅ ***κ***_m_〉. The above results are consistent with the appearance of the MZM at the topological transition. It should be noted that the presence of those MZM implies unconventional *p*-wave superconductivity^8^. As a consequence, for our description to be consistent, the topological phase transition ought to be accompanied by the onset of triplet SC amplitudes Δ_T_. To test this nontrivial effect, we monitored the latter, together with the singlet SC amplitude Δ_S_ (related to a nonlocal *s*-wave SC; see the “Methods” section for detailed definitions). As is evident from the results in Fig. 6, for *U* < *U*_c_ we observe only the singlet component Δ_S_ canonical for an *s*-wave SC, while for *U* ≥ *U*_c_ the triplet amplitude Δ_T_ develops a robust finite value. It is important to stress that Δ_T_ ≠ 0 is an emergent phenomenon, induced by the correlations present within the OSMP, and is not assumed at the level of the model (we use a trivial on-site *s*-wave pairing field as input).

In summary, we have shown that the many competing energy scales induced by the correlation effects present in SC multi-orbital systems within OSMP lead to a topological phase transition. Differently from the other proposed MZM candidate setups, our scheme does not require frozen classical magnetic moments or, equivalently, FM ordering in the presence of the Rashba spin–orbit coupling^3^. All ingredients necessary to host Majorana fermions appear as a consequence of the quantum effects induced by the electron–electron interaction. The pairing filed can be induced by the proximity effect with a BCS superconductor, or it could be an intrinsic property of some iron superconductors under pressure or doping. It is important to note that the coexistence of SC and nontrivial magnetic properties is mostly impossible in single-orbital systems. Here, the OSMP provides a unique platform in which this constraint is lifted by, on the one hand, spatially separating such phenomena, and, on the other hand, strongly correlating them with each other. Furthermore, our proposal allows to study the effect of quantum fluctuations on the MZM modes. There are only a few candidate materials that may exhibit the behavior found here. The block-magnetism (a precursor of the block-spiral phase) was recently argued to be relevant for the chain compound Na_2_FeSe_2_^37^, and was already experimentally found in the BaFe_2_Se_3_ ladder^24^. Incommensurate order was reported in CsFe_2_Se_3_^35^. Also, the OSMP^38–40^ and superconductivity^31–33^ proved to be important for other compounds from the 123 family of iron-based ladders.

Our findings provide also a new perspective to the recent reports of topological superconductivity and Majorana fermions found in two-dimensional compounds Fe(Se,Te)^13–17^. Since orbital-selective features were observed in clean FeSe^41,42^, it is reasonable to assume that OSMP is also relevant for doped Fe(Se,Te)^43^. Regarding magnetism, the ordering of FeSe was mainly studied within the classical long-range Heisenberg model^44^, where block-like structures (e.g., double stripe or staggered dimers) dominate the phase diagram for realistic values of the system parameters. Note that the effective spin model of the block-spiral phase studied here was also argued to be long-ranged^27^. The aforementioned phases of FeSe are typically neighboring (or are even degenerate with) the frustrated spiral-like magnetic orders^44^, also consistent with the OSMP magnetic phase diagram^26^. In view of our results, the following rationale could be used to explain the behavior of the above materials: the competing energy scales present in multi-orbital iron-based compounds, induced by changes in the Hubbard interaction due to chemical substitution or pressure, lead to exotic magnetic spin textures. The latter, together with the SC tendencies, lead to topologically nontrivial phases exhibiting the MZM^45,46^. Also, similar reasoning can be applied to the heavy-fermion metal UTe_2_. It was recently shown that this material displays spin-triplet superconductivity^47^ together with incommensurate magnetism^48^.

## Methods

### DMRG method

The Hamiltonians and observables discussed here were studied using the density matrix renormalization group (DMRG) method^49,50^ within the single-center site approach^51^, where the dynamical correlation functions are evaluated via the dynamical-DMRG^52,53^, i.e., calculating spectral functions directly in frequency space with the correction-vector method using Krylov decomposition^53^. We have kept up to *M* = 1200 states during the DMRG procedures, allowing us to accurately simulate system sizes up to *L* = 48 and *L* = 60 with truncation errors ~10^−8^ and ~10^−6^, respectively.

We have used the DMRG++ computer program developed at Oak Ridge National Laboratory (https://g1257.github.io/dmrgPlusPlus/). The input scripts for the DMRG++ package to reproduce our results can be found at https://bitbucket.org/herbrychjacek/corrwro/ and also on the DMRG++ package webpage.

### Hybrid DMRG–BdG algorithm

We consider a 2D, *s*-wave, BCS superconductor at half-filling,

4 $${H}_{{\rm{BCS}}}=-{t}_{{\rm{BCS}}}\mathop{\sum}\limits_{\langle i,j\rangle ,\sigma }{a}_{i,\sigma }^{\dagger }{a}_{j,\sigma }-{V}_{{\rm{BCS}}}\mathop{\sum}\limits_{i}\left({\Delta }_{i}^{{\rm{BCS}}}\ {a}_{i,\uparrow }^{\dagger }{a}_{i,\downarrow }^{\dagger }+{\rm{H.c.}}\right).$$

Here 〈*i*, *j*〉 denotes summation over nearest-neighbor sites of a square lattice and ${a}_{i,\sigma }^{\dagger }$ (${a}_{i,\sigma }$) creates (destroys) an electron with spin projection *σ* = {↑, ↓} at site *i*. The BCS system is coupled to the OSMP chain, as described by the last term of Hamiltonian (2) in the main text. At the BCS level, the latter term emerges as an additional (external) pairing field to the OSMP region

5 $${H}_{V}=-V\mathop{\sum}\limits_{{\ell }^{\prime}}\left({\Delta }_{\ell }^{{\rm{OSMP}}}\ {a}_{{\ell }^{\prime},\uparrow }^{\dagger }{a}_{{\ell }^{\prime},\downarrow }^{\dagger }+{\rm{H.c.}}\right).$$

Here, the summation is restricted to the sites of the BCS system which are coupled to the OSMP chain. In numerical calculations, we set the hopping integral *t*_BCS_ = 2 [eV], fix the system size to *L*_*x*_ = 54 and *L*_*y*_ = 27 (with 1D OSMP system coupled to the ${\ell }^{\prime}=14$ row of sites), use the BCS attractive potential *V*_BCS_/*t*_BCS_ = 2 and the coupling strength *V*/*t*_BCS_ = 2. Although we assume periodic boundary conditions for the BCS system, the translational invariance is broken by the coupling to the OSMP chain.

Our procedure consists of two alternating steps:

1. *BdG calculations*: In the first step, we assume an initial set ${\Delta }_{\ell }^{{\rm{OSMP}}}$ and diagonalize the Hamiltonian *H*_BCS_ + *H*_*V*_, as defined in Eqs. (4) and (5). To this end, we use the standard BdG equations at zero temperature. They yield self-consistent results for the pairing amplitude, ${\Delta }_{i}^{{\rm{BCS}}}=\langle {a}_{i,\downarrow }{a}_{i,\uparrow }\rangle$, for all sites *i* within the BCS system. From among the latter results, we single out the amplitudes ${\Delta }_{{\ell }^{\prime}}^{{\rm{BCS}}}$ on the sites $i={\ell }^{\prime}$ which are coupled to the OSMP chain.
2. *DMRG calculations*: The OSMP system within Eq. (3) is evaluated using the DMRG approach. The spatially dependent amplitudes ${\Delta }_{\ell }^{{\rm{OSMP}}}$ are calculated providing a new set of external fields for the subsequent BdG calculations.

The above procedure is repeated iteratively until we obtain converged results. In the main text (see Fig. 2) we presented results of the above algorithm starting from ${\Delta }_{\ell }^{{\rm{OSMP}}}=0$. However, the procedure can also start from arbitrary pairing fields ${\Delta }_{\ell }^{{\rm{OSMP}}}$ in the first step. The right column of Fig. 2b depicts results obtained using a random initial profile ${\Delta }_{\ell }^{{\rm{OSMP}}}\in [0,1]$. It is evident from the presented results that the converged result is independent of the initial configuration (at least for the couplings studied here). See Supplementary Note 1 for further discussion and additional results.

### Spectral functions

Let us define the site-resolved frequency (*ω*)-dependent electron (e) and hole (h) correlation functions

6 $${\langle \langle {A}_{\ell }\ {B}_{m}\rangle \rangle }_{\omega }^{{\rm{e}},{\rm{h}}}=-\frac{1}{\pi }{\rm{Im}}\left\langle {\rm{gs}}\right|{A}_{\ell }\frac{1}{{\omega }^{+}\mp (H-{\epsilon }_{0})}{B}_{m}\left|{\rm{gs}}\right\rangle ,$$

where the signs + and − should be taken for ${\langle \langle ...\rangle \rangle }_{\omega }^{\rm{{{e}}}}$ and ${\langle \langle}\! {\ldots}\!\!{\rangle \rangle }_{\omega }^{\rm{{{h}}}}$, respectively. Here, $\left|{\rm{gs}}\right\rangle$ is the ground-state, *ϵ*_0_ the ground-state energy, and *ω*^+^ = *ω* + *iη* with *η* a Lorentzian-like broadening. For all results presented here, we choose *η* = 2*δω*, with *δω*/*W* = 0.001 (unless stated otherwise).

The single-particle spectral functions *A*(*q*, *ω*) = *A*^e^(*q*, *ω*) + *A*^h^(*q*, *ω*), where *A*^e^ (*A*^h^) represent the electron (hole) part of the spectrum, have a standard definition,

7 $$\begin{array}{lll}{A}^{{\rm{h}}}(q,\omega )&=&\mathop{\sum}\limits_{\ell }{{\rm{e}}}^{-iq(\ell -L/2)}\ {\langle \langle {c}_{\ell }\ {c}_{L/2}^{\dagger }\rangle \rangle }_{\omega }^{{\rm{h}}},\\ {A}^{{\rm{e}}}(q,\omega )&=&\mathop{\sum}\limits_{\ell }{{\rm{e}}}^{+iq(\ell -L/2)}\ {\langle \langle {c}_{\ell }^{\dagger }\ {c}_{L/2}\rangle \rangle }_{\omega }^{{\rm{e}}},\end{array}$$

with ${c}_{\ell }={\sum }_{\sigma }{c}_{\ell ,\sigma }$. Finally, the LDOS is defined as

8 $${\rm{LDOS}}(\ell ,\omega )={\langle \langle {c}_{\ell }\ {c}_{\ell }^{\dagger }\rangle \rangle }_{\omega }^{{\rm{h}}}+{\langle \langle {c}_{\ell }^{\dagger }\ {c}_{\ell }\rangle \rangle }_{\omega }^{{\rm{e}}}.$$

### Spectral functions of Majorana edge-states

For simplicity, in this section, we suppress the spin index *σ* and assume that the lattice index *j* contains all local quantum numbers. The many-body Hamiltonian is originally expressed in terms of fermionic operators ${c}_{j}^{\left(\dagger \right)}$, but it may be equivalently rewritten using the Majorana fermions (not to be confused with the MZM):

9 $${\gamma }_{2j-1}={c}_{j}+{c}_{j}^{\dagger },\quad \quad {\gamma }_{2j}=-i({c}_{j}-{c}_{j}^{\dagger }),$$

where ${\gamma }_{l}^{\dagger }={\gamma }_{l}$ and {*γ*_*i*_, *γ*_*j*_} = 2*δ*_*ij*_. The latter anticommutation relation is invariant under orthogonal transformations, thus we can rotate the Majorana fermions arbitrarily with

10 $${{{\Gamma }}}_{a}=\mathop{\sum}\limits_{j}{\hat{V}}_{aj}{\gamma }_{j},$$

where $\hat{V}$ are real, orthogonal matrices ${\hat{V}}^{\top }\hat{V}=\hat{V}{\hat{V}}^{\top }=1$. If the system hosts a pair of Majorana edge modes, Γ_L_ and Γ_R_, then we can find a transformation $\hat{V}$ such that the following Hamiltonian captures the low-energy physics

11 $$H\simeq \ i\frac{\varepsilon }{2}\ {{{\Gamma }}}_{{\rm{{L}}}}\ {{{\Gamma }}}_{{\rm{{R}}}}+H^{\prime} .$$

It is important to note that $H^{\prime}$ does not contribute to the in-gap states. It contains all Majorana operators, Γ_a_, other than the MZM (Γ_L_ and Γ_R_). The first term in Eq. (11) arises from the overlap of the MZM in a finite system, while in the thermodynamic limit *ε* → 0 both Γ_L_ and Γ_R_ become strictly the zero modes. While the ground state properties obtained from the zero temperature DMRG do not allow us to formally construct the transformation $\hat{V}$, we demonstrate below that the computed local and non-local spectral functions are fully consistent with the MZM. In fact, we are not aware of any other scenario that could explain the spectral functions reported in this work.

Let us investigate the retarded Green’s functions

12 $$\begin{array}{lll}{G}^{{\rm{h}}}\left({c}_{j},{c}_{l}^{\dagger }\right)&=&-i\mathop{\int }\limits_{0}^{\infty }{\rm{d}}t\ {e}^{i\omega t}\left\langle {\rm{gs}}\right|{c}_{j}(t){c}_{l}^{\dagger }\left|{\rm{gs}}\right\rangle ,\\ {G}^{{\rm{e}}}\left({c}_{j},{c}_{l}^{\dagger }\right)&=&-i\mathop{\int }\limits_{0}^{\infty }{\rm{d}}t\ {e}^{i\omega t}\left\langle {\rm{gs}}\right|{c}_{l}^{\dagger }{c}_{j}(t)\left|{\rm{gs}}\right\rangle ,\end{array}$$

which are related to the already introduced spectral functions

13 $$\begin{array}{l}{\langle \langle {c}_{j}{c}_{l}^{\dagger }\rangle \rangle }_{\omega }^{{\rm{h}}}=-\frac{1}{\pi }{\rm{Im}}\ {G}^{{\rm{h}}}\left({c}_{j},{c}_{l}^{\dagger }\right),\\ {\langle \langle {c}_{l}^{\dagger }{c}_{j}\rangle \rangle }_{\omega }^{{\rm{e}}}=-\frac{1}{\pi }{\rm{Im}}\ {G}^{{\rm{e}}}\left({c}_{j},{c}_{l}^{\dagger }\right).\end{array}$$

Using the transformations (9) and (10) one may express ${G}^{{\rm{e}},{\rm{h}}}\left({c}_{j},{c}_{l}^{\dagger }\right)$ as a linear combination of the Green’s functions defined in terms of the Majorana fermions ${G}^{{\rm{e}},{\rm{h}}}\left({{{\Gamma }}}_{a},{{{\Gamma }}}_{b}\right)$. However, the only contributions to the in-gap spectral functions come from the zero-modes, i.e., from *a*, *b* ∈ {L, R}, and the corresponding functions can be obtained directly from the effective Hamiltonian (11),

14 $$\begin{array}{lll}{G}^{{\rm{h}}}\left({{{\Gamma }}}_{{\rm{{L}}}},{{{\Gamma }}}_{{\rm{{L}}}}\right)={G}^{{\rm{h}}}\left({{{\Gamma }}}_{{\rm{{R}}}},{{{\Gamma }}}_{{\rm{{R}}}}\right)&=&\frac{1}{\omega -| \varepsilon | +i\eta },\\ {G}^{{\rm{e}}}\left({{{\Gamma }}}_{{\rm{{L}}}},{{{\Gamma }}}_{{\rm{{L}}}}\right)={G}^{{\rm{e}}}\left({{{\Gamma }}}_{{\rm{{R}}}},{{{\Gamma }}}_{{\rm{{R}}}}\right)&=&\frac{1}{\omega +| \varepsilon | +i\eta }.\end{array}$$

The Green’s functions determine the in-gap peak in the left part of the system

15 $${G}^{\alpha }\left({c}_{j},{c}_{j}^{\dagger }\right)=\frac{{V}_{{\rm{{L}}},2j}^{2}+{V}_{{\rm{{L}}},2j-1}^{2}}{4}\ {G}^{\alpha }\left({{{\Gamma }}}_{{\rm{{L}}}},{{{\Gamma }}}_{{\rm{{L}}}}\right),$$

with *α* ∈ {e, h}, and a similar expression holds for the peak on its right side. Utilizing the orthogonality of $\hat{V}$, one may explicitly sum up the Green’s functions over the lattice sites

16 $$\mathop{\sum}\limits_{j}{G}^{\alpha }\left({c}_{j},{c}_{j}^{\dagger }\right)=\frac{1}{4}{G}^{\alpha }\left({{{\Gamma }}}_{{\rm{{L}}}},{{{\Gamma }}}_{{\rm{{L}}}}\right),$$

where the sum over *j* contains few sites at the edge of the system due to the exponential decay of the $\hat{V}$ elements. The result Eq. (16) explains why the total spectral weights originating from ∑_*j*_*G*^*α*^ equal 1/4, while the total spectral weights of the peaks in LDOS equal 1/2. A similar discussion of the nonlocal centrosymmetric spectral functions ${\langle \langle {c}_{\ell }\ {c}_{L-\ell +1}^{\dagger }\rangle \rangle }_{{\epsilon }_{{\rm{F}}}}^{{\rm{h}}}$ and ${\langle \langle {c}_{\ell }^{\dagger }\ {c}_{L-\ell +1}\rangle \rangle }_{{\epsilon }_{{\rm{F}}}}^{{\rm{e}}}$ can be found in Supplementary Note 3.

### Von Neumann entanglement entropy

*S*_vN_(*ℓ*) measures entanglement between two subsystems containing, respectively, *ℓ* and *L*−*ℓ* sites, and can be easily calculated within DMRG via the reduced density matrix *ρ*_*ℓ*_, i.e., ${S}_{{\rm{vN}}}(\ell )=-{\rm{Tr}}{\rho }_{\ell }{\mathrm{ln}}\,{\rho }_{\ell }$. The results presented in Fig. 6 depict the system divided into two equal halves, *ℓ* = *L*/2. The full spatial dependence of *S*_vN_(*ℓ*) is presented in Supplementary Note 5.

### SC amplitudes

The *s*-wave and *p*-wave SC can be detected with singlet Δ_S_ and triplet Δ_T_ amplitudes, respectively, defined as

17 $${\Delta }_{{\rm{S}}}= \mathop{\sum }\limits_{\ell =L/4}^{3L/4}\left|{\Delta }_{{\rm{S}},\ell }\right|,\\ {\Delta }_{{\rm{T}}}= \mathop{\sum }\limits_{\ell =L/4}^{3L/4}\left(\left|{\Delta }_{{\rm{T0}},\ell }\right|+\left|{\Delta }_{{\rm{T\downarrow }},\ell }\right|+\left|{\Delta }_{{\rm{T\downarrow }},\ell }\right|\right),$$

with

18 $${\Delta }_{{\rm{S}},\ell } =\left\langle {c}_{\ell ,\uparrow }^{\dagger }{c}_{\ell +1,\downarrow }^{\dagger }-{c}_{\ell ,\downarrow }^{\dagger }{c}_{\ell +1,\uparrow }^{\dagger }\right\rangle ,\\ {\Delta }_{{\rm{T0}},\ell } =\left\langle {c}_{\ell ,\uparrow }^{\dagger }{c}_{\ell +1,\downarrow }^{\dagger }+{c}_{\ell ,\downarrow }^{\dagger }{c}_{\ell +1,\uparrow }^{\dagger }\right\rangle ,\\ {\Delta }_{{\rm{T\uparrow }},\ell } =\left\langle {c}_{\ell ,\uparrow }^{\dagger }{c}_{\ell +1,\uparrow }^{\dagger }\right\rangle ,\quad {\Delta }_{{\rm{T\downarrow }},\ell }=\left\langle {c}_{\ell ,\downarrow }^{\dagger }{c}_{\ell +1,\downarrow }^{\dagger }\right\rangle .$$

## Supplementary information

Supplementary Information

## Data Availability

The data that support the findings of this study are available from the corresponding author upon request.
