# Supplementary material for: Interaction-induced topological phase transition and Majorana edge states in low-dimensional orbital-selective Mott insulators
Source: Nat Commun. 2021 May 19;12:2955. doi: 10.1038/s41467-021-23261-2 (PMC8134496; doi:10.1038/s41467-021-23261-2)
Supplement: Supplementary file 1 — Supplementary Information [file 41467_2021_23261_MOESM1_ESM.pdf]

**SUPPLEMENTARY INFORMATION** for:

# Interaction-induced topological phase transition and Majorana edge states in low-dimensional orbital-selective Mott insulators

by J. Herbrych, M. Środa, G. Alvarez, M. Mierzejewski, and E. Dagotto

## CONTENTS

Supplementary Note 1. Stability of the hybrid BdG-DMRG algorithm 2

Supplementary Note 2. Ladder geometry considerations 3

Supplementary Note 3. Spectral functions 4

Supplementary Note 4. Parameter dependence 6

Supplementary Note 5. Entropy and dimer order 10

Supplemental References 12

## Supplementary Note 1. Stability of the hybrid BdG-DMRG algorithm

In the main text, we have presented how the spatial profiles of the pairing fields (PF) in the BCS system $\Delta_i^{\rm BCS}$ converge in subsequent steps of the iteration procedure. Here, as supplementary information, we will discuss the convergence of PF $\Delta_\ell^{\rm OSMP}$ obtained for the 1D OSMP system. Furthermore, we will test the stability of the introduced procedure and demonstrate that the obtained results are independent of the initial choice of the PF.

From the results presented in the main text, it is evident that the PF converge to almost uniform (spatially independent) values within the 1D OSMP system. Consequently, to simplify the presentation of the results, we will discuss only the behaviour of the average PF, $\sum_\ell \Delta_\ell^{\rm OSMP}/L$. In Supplementary Figure 1 we present the iteration dependence of the latter for various initial starting points. As explained in the Methods section of the main text, our DMRG-BdG algorithm can be started from arbitrary amplitudes in the 1D OSMP system. We have considered: (1) zero PF (the result discussed in the main text), (2) constant PF $\Delta_\ell^{\rm OSMP}=0.1$ and $\Delta_\ell^{\rm OSMP}=1.0$, and (3) random PF drawn from a box distribution of widths $[0.0\,,0.1]$, $[0.0\,,1.0]$, $[-0.1\,,0.1]$, and $[-1.0\,,0.1]$. Several conclusions can be obtained directly from the results for $U/W=1$ (trivial phase, Supplementary Figure 1a) and $U/W=2$ (topological phase, Supplementary Figure 1b): (i) For all considered cases, the converged spatial profiles are almost uniform within the OSMP chain. This is best exemplified by the results presented in the right column of Fig. 2b of the main text, where we show the convergence of the hybrid procedure for the case when iterations are initialized by random PF, i.e., $\Delta_\ell^{\rm OSMP}\in[0,1]$. (ii) For all considered initial PF, the results converge to the same - interaction dependent - value. This nontrivial result shows that the hybrid BdG-DMRG procedure is numerically very stable. (iii) The quick convergence of the hybrid procedure holds true for the entire range of the Coulomb repulsion considered in the present studies: see results for $\sum_\ell \Delta_\ell^{\rm OSMP}/L$ shown in Supplementary Figure 1c and Supplementary Figure 1d.

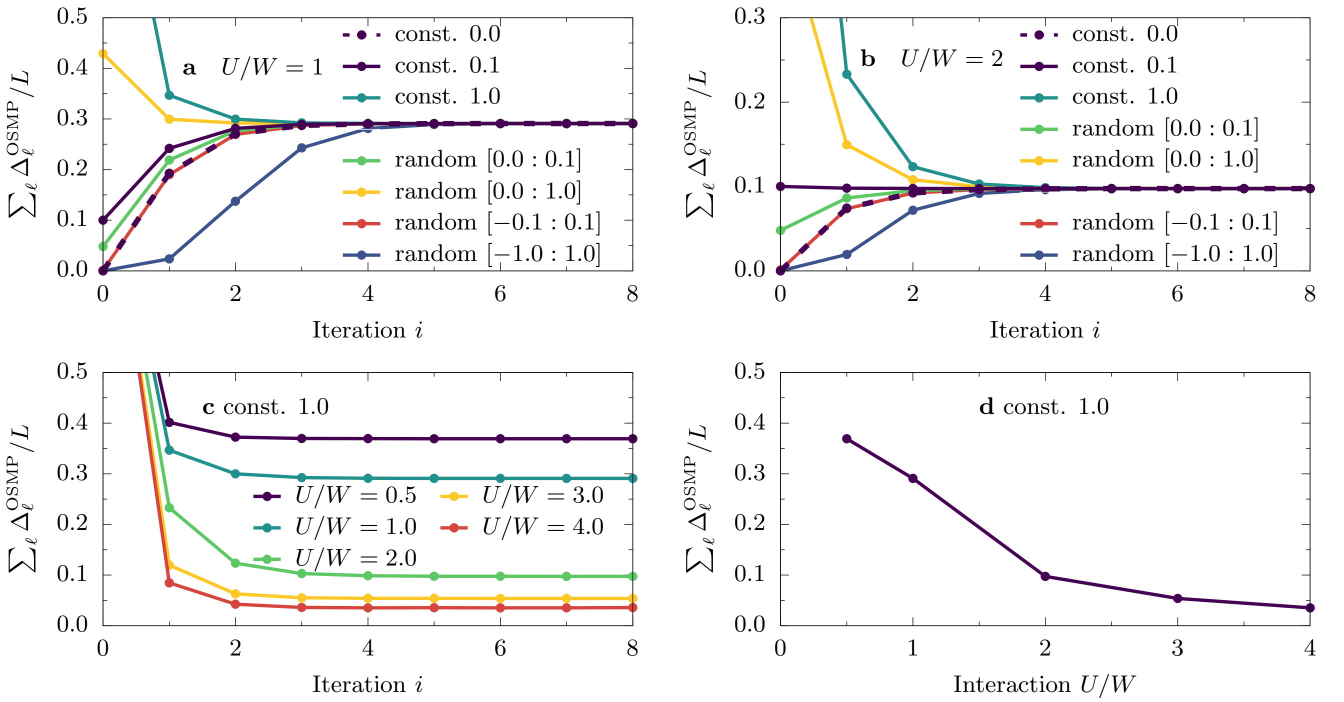


Supplementary Figure 1. **Pairing fields within the 1D OSMP system. a,b** Iteration convergence of the average pairing field amplitudes $\sum_\ell \Delta_\ell^{\rm OSMP}/L$ as obtained for **a** $U/W=1$ and **b** $U/W=2$, starting from various initial configuration of $\Delta_\ell^{\rm OSMP}$. Calculated for $L=36$, $\overline{n}=0.5$, and $V=4\,[\rm eV]$. All units in eV. See the Methods section of the main text for details. **c** depicts the interaction $U$ dependence of the convergence for the case of an initial $\Delta_\ell^{\rm OSMP}=1\,[\rm eV]$. The asymptotic value of $\sum_\ell \Delta_\ell^{\rm OSMP}/L$ as function of $U$ is given in **d**.

## Supplementary Note 2. Ladder geometry considerations

In the main text, we have shown that the generalized Kondo-Heisenberg (gKH) model on the chain geometry can support Majorana zero-energy modes (MZM) when in the presence of a superconducting (SC) pairing field $\Delta_{\mathrm{SC}}$. Here, we will show that the key ingredients necessary to support the MZM in the gKH model are also present in the ladder geometry, i.e. the block-spiral magnetic state (see Supplementary Figure 2a for a sketch) and single-particle spectra with parity-breaking quasi-particles. We will consider a spatially isotropic ladder with $t_{\parallel} = t_{\perp} \equiv t_{\mathrm{i}}$, the latter hopping defined in the main text, and choose filling $\overline{n} = 1.75$, which supports block-magnetism at $U \sim W$ [1, 2]. Although accurate calculations within the grand-canonical ensemble (needed with finite pairing field $\Delta_{\mathrm{SC}} \neq 0$) are numerically too demanding (due to the doubling of the lattice sites on the ladder of $L$ rungs), precise canonical calculations of the static structure factor can still be performed, i.e.,

$$S(q_{\parallel}, q_{\perp}) = \sum_{\ell,\ell'} \sum_{r,r'} \mathrm{e}^{+iq_{\perp}(r-r')} \mathrm{e}^{+iq_{\parallel}(\ell-\ell')} \langle \mathbf{T}_{\ell,r} \cdot \mathbf{T}_{\ell',r'} \rangle \,, \tag{1}$$

where $\mathbf{T}_{\ell,r} = \mathbf{S}_{\ell,r} + \mathbf{s}_{\ell,r}$, and $(\ell, r)$ represent the leg and rung number, respectively. Our results in Supplementary Figure 2b reveal that the $S(q_{\parallel}, q_{\perp})$ lies at incommensurate values of the wavevectors, the one of the block-spiral magnetic state signatures [3]. Another feature of the latter is the existence of two cosine-like bands in the single-particle spectral (see also the discussion in the next section) $A(q_{\parallel}, q_{\perp}, \omega) = A^{\mathrm{e}}(q_{\parallel}, q_{\perp}, \omega) + A^{\mathrm{h}}(q_{\parallel}, q_{\perp}, \omega)$ near the Fermi level $\omega \sim \epsilon_{\mathrm{F}}$, where

$$\begin{aligned} A^{\mathrm{e}}(q_{\parallel}, q_{\perp}, , \omega) &= \sum_{\ell} \sum_{r,r'} \mathrm{e}^{+iq_{\perp}(r-r')} \mathrm{e}^{-iq_{\parallel}(\ell-L/2)} \, \langle\langle c_{\ell,r} \, c^{\dagger}_{L/2,r'} \rangle\rangle^{\mathrm{e}}_{\omega} \,, \\ A^{\mathrm{h}}(q_{\parallel}, q_{\perp}, , \omega) &= \sum_{\ell} \sum_{r,r'} \mathrm{e}^{+iq_{\perp}(r-r')} \mathrm{e}^{+iq_{\parallel}(\ell-L/2)} \, \langle\langle c^{\dagger}_{\ell,r} \, c_{L/2,r'} \rangle\rangle^{\mathrm{h}}_{\omega} \,. \end{aligned} \tag{2}$$

The results presented in Supplementary Figure 2c (for $L = 36$ rungs, $U/W = 2.2$, $J_{\mathrm{H}}/U = 0.25$, and $\overline{n} = 1.75$) are consistent with this scenario and resemble the chain geometry results. Consequently, it is reasonable to assume that the influence of a finite pairing field $\Delta_{\mathrm{SC}} \neq 0$ will lead to a topological superconducting state and the emergence of the MZM also on the ladder lattice.

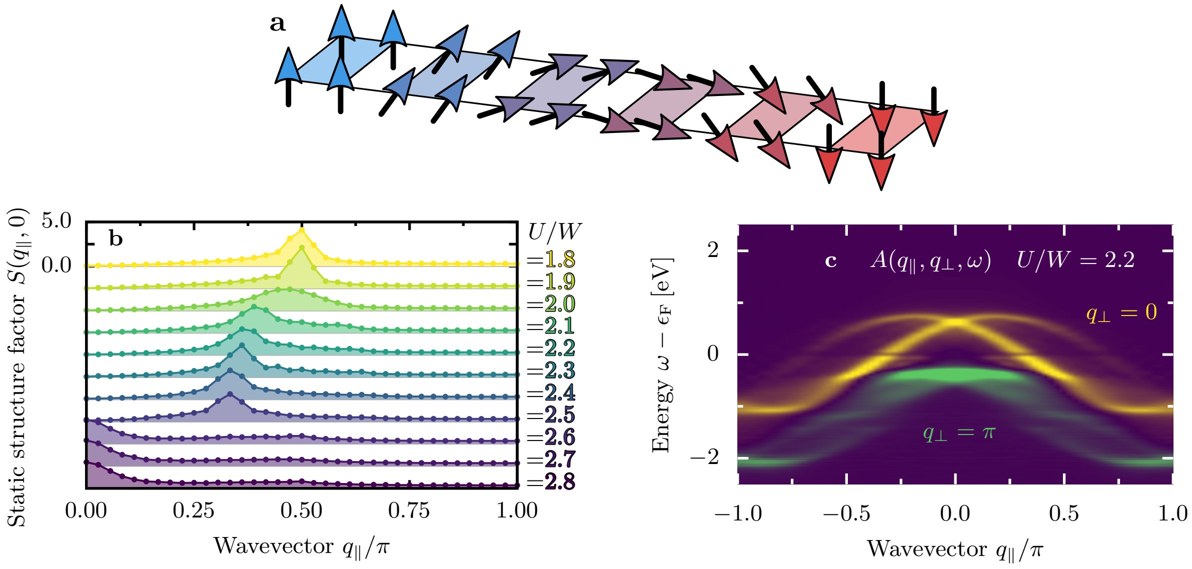


Supplementary Figure 2. **Block-spiral state on the ladder geometry.** **a** Sketch of the block-spiral state on the ladder geometry. Color (shaded) area represents rigidly rotating $2 \times 2$ FM blocks. **b** Interaction dependence of the symmetric component $q_{\perp} = 0$ of the static structure factor $S(q_{\parallel}, q_{\perp} = 0)$. **c** Wavevector dependence of both components (symmetric and antisymmetric) of the single-particle spectral function $A(q_{\parallel}, q_{\perp} = 0, \omega) + A(q_{\parallel}, q_{\perp} = \pi, \omega)$ near the Fermi level in the block-spiral phase ($\eta = 2\delta\omega$ and $\delta\omega = 0.02$ [eV]). All results calculated for the gKH ladder of $L = 36$ rungs, $J_{\mathrm{H}}/U = 0.25$, $\overline{n} = 1.75$, and $\Delta_{\mathrm{SC}} = 0$.

## Supplementary Note 3. Spectral functions

In Fig. 4 of the main text, we have shown the spatial dependence of the local density-of-states (LDOS) at the Fermi level, together with equal contributions of the electron and hole components, as expected for MZM. Here, in Supplementary Figure 3, we show that the same holds in frequency $\omega$ space. Furthermore, in the same figure we show that both spin components contribute equally (within our numerical precision).

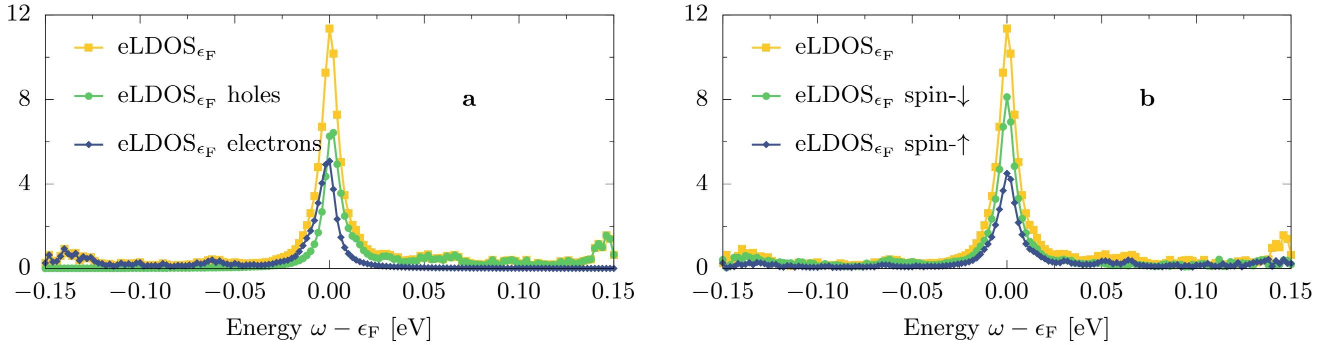


Supplementary Figure 3. **Components of the Majorana edge states.** Frequency $\omega$ dependence of the edge-LDOS ($\ell = 1$) near the Fermi level $\omega \sim \epsilon_F$ Panel **a** depicts electron and hole contributions, while panel **b** the ↓- and ↑-spin component. Calculated for $L = 36$, $U/W = 2$, $\Delta_{SC}/W \simeq 0.5$, and $\overline{n} = 0.5$.

The same reasoning used for the LDOS in the main text can also be applied to the off-diagonal functions $G^{\alpha}\left(c_j, c_l^{\dagger}\right)$ where sites $j$ and $l$ belong to the left $j < L/2$ and the right $l > L/2$ portions of the system. Then, it can be shown that

$$G^{\alpha}\left(c_j, c_l^{\dagger}\right) = \frac{V_{L,2j-1}V_{R,2l-1} + V_{L,2j}V_{R,2l}}{4} G^{\alpha}\left(\Gamma_L, \Gamma_R\right), + i\frac{V_{L,2j}V_{R,2l-1} - V_{L,2j-1}V_{R,2l}}{4} G^{\alpha}\left(\Gamma_L, \Gamma_R\right), \quad (3)$$

with

$$G^{\mathrm{h}}\left(\Gamma_L, \Gamma_R\right) = -G^{\mathrm{h}}\left(\Gamma_R, \Gamma_L\right) = \frac{i\,\mathrm{sgn}(\varepsilon)}{\omega - |\varepsilon| + i\eta}, \quad G^{\mathrm{e}}\left(\Gamma_L, \Gamma_R\right) = -G^{\mathrm{e}}\left(\Gamma_R, \Gamma_L\right) = \frac{-i\,\mathrm{sgn}(\varepsilon)}{\omega + |\varepsilon| + i\eta}. \quad (4)$$

Since the considered Hamiltonian is real, the spectral functions $\langle\langle c_j c_l^{\dagger}\rangle\rangle_{\omega}^{\mathrm{h}}$ and $\langle\langle c_j^{\dagger} c_l\rangle\rangle_{\omega}^{\mathrm{e}}$ should be real as well. Given that the weights of $\langle\langle \Gamma_L \Gamma_R\rangle\rangle_{\omega}^{\alpha}$ are purely imaginary [see Supplementary Eq. (4)], the upper line in Supplementary Eq. (3) should vanish. Indeed, for real Hamiltonians, $\Gamma_L$ (and also $\Gamma_R$) contains $\gamma_j$ with only even or odd $j$. In other words, $\Gamma_L$ contains only $\gamma_{2j}$ and $\Gamma_R$ contains only $\gamma_{2j-1}$ or *vice versa*. Without losing generality, we may choose the former possibility,

$$G^{\alpha}\left(c_j, c_l^{\dagger}\right) = i\frac{V_{L,2j}V_{R,2l-1}}{4} G^{\alpha}\left(\Gamma_L, \Gamma_R\right) \quad G^{\alpha}\left(c_l, c_j^{\dagger}\right) = -i\frac{V_{R,2l-1}V_{L,2j}}{4} G^{\alpha}\left(\Gamma_R, \Gamma_L\right) = G^{\alpha}\left(c_j, c_l^{\dagger}\right), \quad (5)$$

and obtain the spectral functions shown in Supplementary Figure 4 and Fig. 4b of the main text

$$\langle\langle c_l c_{L-l+1}^{\dagger}\rangle\rangle_{\omega}^{\mathrm{h}} = -\frac{1}{4}\left\{\begin{array}{ll} V_{L,2l}V_{R,2L-2l+1} & \text{for } l < L/2 \\ V_{L,2L-2l+2}V_{R,2l-1} & \text{for } l > L/2 \end{array}\right\} \mathrm{sgn}(\varepsilon)\,\delta(\omega - |\varepsilon|), \quad \langle\langle c_l^{\dagger} c_{L-l+1}\rangle\rangle_{\omega}^{\mathrm{e}} = +\frac{1}{4}\left\{\begin{array}{ll} V_{L,2l}V_{R,2L-2l+1} & \text{for } l < L/2 \\ V_{L,2L-2\ell+2}V_{R,2l-1} & \text{for } l > L/2 \end{array}\right\} \mathrm{sgn}(\varepsilon)\,\delta(\omega + |\varepsilon|), \quad (6)$$

where the electron and hole contributions arise with opposite signs, as it is also visible in Supplementary Figure 4 and Fig. 4b of the main text. Finally, it is reasonable to assume that the spatial profiles of the MZMs at both

system edges are mutually symmetric, i.e., $|V_{R,2L-2l+1}| \simeq |V_{L,2l}|$. Then, comparing Eq. 15 of the main text and Supplementary Eq. (6) we obtain a mirroring of the diagonal (local) and off-diagonal spectral functions

$$\begin{aligned} |\langle\langle c_l\, c^\dagger_{L-l+1}\rangle\rangle^{\rm h}_\omega| &\simeq |\langle\langle c_l\, c^\dagger_l\rangle\rangle^{\rm h}_\omega|, \\ |\langle\langle c^\dagger_{L-l+1} c_l\rangle\rangle^{\rm e}_\omega| &\simeq |\langle\langle c^\dagger_l c_l\rangle\rangle^{\rm e}_\omega|, \end{aligned} \tag{7}$$

which is reasonably well reproduced by the numerical results.

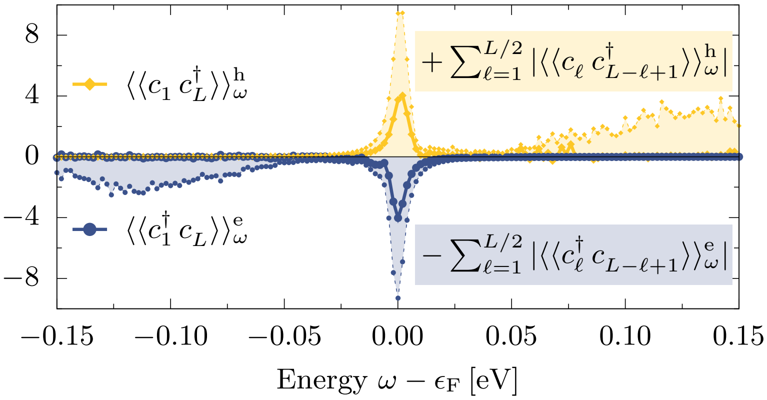


Supplementary Figure 4. **Off-diagonal spectral functions.** Frequency dependence of centrosymmetric spectral functions, Supplementary Eq. (6), at the edge of the system $\ell = 1$ (solid points). We also present spatially integrated, according to Eq. 16 of the main text, spectral functions as colored area. Results shown were calculated for $L = 36$, $U/W = 2$, $\Delta_{\rm SC}/W \simeq 0.5$, and $\overline{n} = 0.5$.

## Supplementary Note 4. Parameter dependence

In this section, we will discuss the pairing field $\Delta_{\rm SC}$ dependence of our results. Let us first focus on the single-particle spectral function $A(q,\omega)$ [see Eq. 7 of the main text]. In Supplementary Figure 5a and Supplementary Figure 5b we show $A(q,\omega)$ for systems (at electronic filling $\overline{n}=0.5$) without pairing field $\Delta_{\rm SC}=0$ for two representative values of the interaction: $U/W=1$ and $U/W=2$, i.e., in the block-collinear and block-spiral magnetic phases. Both spectra exhibit a finite density-of-states (DOS) at the Fermi level $\epsilon_{\rm F}$. In the case of the block-spiral phase at $U/W=2$, Supplementary Figure 5b, one can observe two bands of quasiparticles: left and right movers reflecting the two possible rotations of the spirals. It is obvious from these results that the quasiparticles break the parity symmetry; i.e., going from $q\to -q$ momentum changes the quasiparticle character, as expected for a spiral state. It is also worth noting that for the block-magnetic order ($U/W=1$ and $\Delta_{\rm SC}=0$) one can observe [3] the V-like shape of DOS in the vicinity of $\epsilon_{\rm F}$. The latter indicates a semiconductor-like behaviour which was also experimentally found [4] in the $2\times 2$ block-magnetic ladder compound $\rm BaFe_2Se_3$. This result shows our model's strength and relevance for realistic investigations of the iron-based materials from the 123 family.

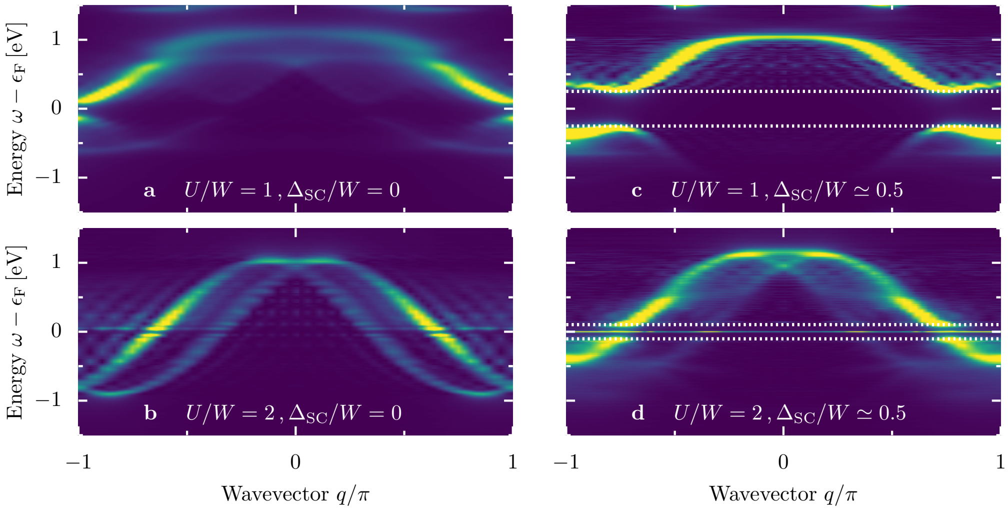


Supplementary Figure 5. **Single-particle spectra for block-collinear and block-spiral magnetism.** Single-particle spectra $A(q,\omega)$ of the gKH model using $L=36$ sites and electronic filling $\overline{n}=0.5$ for **a** $U/W=1\,,\Delta_{\rm SC}=0$, **b** $U/W=2\,,\Delta_{\rm SC}=0$, **c**, $U/W=1\,,\Delta_{\rm SC}/W\simeq 0.5$, and **d** $U/W=2\,,\Delta_{\rm SC}/W\simeq 0.5$.

The pairing field $\Delta_{\rm SC}\neq 0$ has a striking effect on these two phases, see Supplementary Figure 5c and Supplementary Figure 5d where we present results for $\Delta_{\rm SC}/W\simeq 0.5$. As discussed in detail in the main text, the pairing field leads to the appearance of MZM in the spiral phase ($U/W=2$, Supplementary Figure 5d), with the flat $\delta$-mode inside the superconducting gap. On the other hand, for the collinear block-magnetic phase ($U/W=1$, Supplementary Figure 5c) we observe only a trivial opening of the SC gap, without any in-gap states. These results indicate that the Hubbard interaction strength $U$, as the main driver between the two magnetic states, plays a crucial role in the stabilization of the MZM.

In order to investigate all these aspects in more detail, in Supplementary Figure 6 we present the pairing field $\Delta_{\rm SC}$ dependence of the quantities discussed in the main text, i.e.: (i) value of edge-LDOS at the Fermi level $\epsilon_{\rm F}$ (eLDOS), (ii) chirality correlation function $\langle\boldsymbol{\kappa}_{L/4}\cdot\boldsymbol{\kappa}_{3L/4}\rangle$ at $L/2$ distance ($\boldsymbol{\kappa}_{L/2}$), and (iii) amplitudes of extended (non-local) SC singlet and triplet amplitudes, $\Delta_{\rm S}$ and $\Delta_{\rm T0}$, respectively [see Eq. 17 of the main text]. Furthermore, in the same figure we present the value of the on-site pairing amplitude

$$\Delta_0=\frac{2}{L}\sum_{\ell=L/4}^{3L/4}\left|c^{\dagger}_{\ell,\uparrow}c^{\dagger}_{\ell,\downarrow}\right|\,. \tag{8}$$

For the collinear block-magnetic phase ($U/W=1$, Supplementary Figure 6a) we observe that the $\Delta_{\rm SC}$ does not induce any topological phase transitions. For all considered values of the pairing field, $0<\Delta_{\rm SC}/W\lesssim 0.7$, the eLDOS,

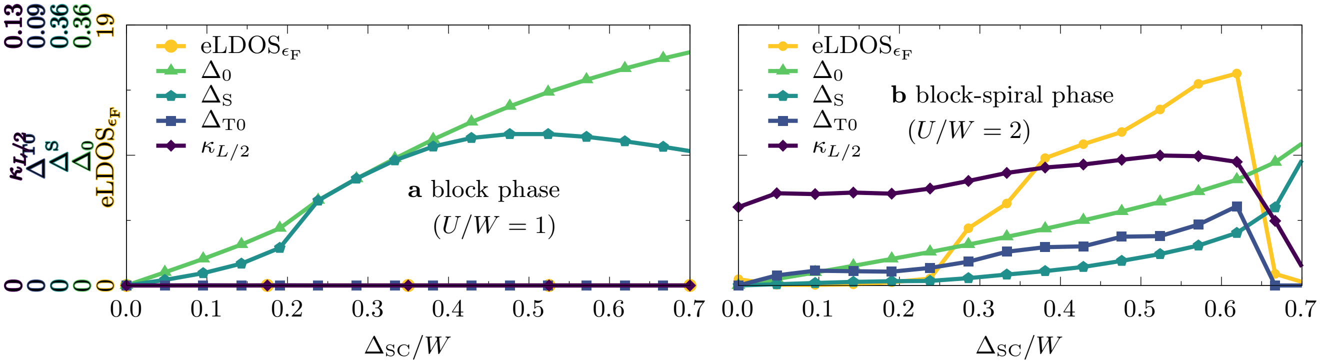


Supplementary Figure 6. **Phase diagram.** Pairing field $\Delta_{SC}$ dependence of: (i) the value of edge-LDOS at the Fermi level $\epsilon_F$ (eLDOS), (ii) chirality correlation function $\langle \boldsymbol{\kappa}_{L/4} \cdot \boldsymbol{\kappa}_{3L/4} \rangle$ at $L/2$ distance ($\boldsymbol{\kappa}_{L/2}$), (iii) amplitudes of local and non-local SC singlet amplitudes, $\Delta_0$ and $\Delta_S$, respectively, together with triplet component $\Delta_{T0}$. Panel **a** shows results for the block-collinear magnetic phase ($U/W = 1$), while panel **b** for the block-spiral phase ($U/W = 2$). All results were calculated for $L = 36$ and $\overline{n} = 0.5$.

$\boldsymbol{\kappa}_{L/2}$, and $\Delta_T$ are zero. Only the singlet SC amplitudes, local $\Delta_0$ and non-local $\Delta_S$, take a finite value. The behaviour of the $U/W = 2$ case is strikingly different (see Supplementary Figure 6b). The chirality correlation function $\boldsymbol{\kappa}_{L/2}$ has a finite value already at $\Delta_{SC} = 0$, reflecting the block-spiral ordering at this interaction strength, and weakly changes till $\Delta_{SC}/W \sim 0.6$, after which it decays to zero. Simultaneously, the triplet SC amplitude $\Delta_{T0}$ increases smoothly with the pairing-field $\Delta_{SC}$ (together with singlet components $\Delta_0$ and $\Delta_S$). It is worth noting that this behaviour is strikingly different from the $U$ variation presented in the main text, where we observed a sudden appearance of $\boldsymbol{\kappa}_{L/2}$ and $\Delta_{T0}$ at a specific value of $U_c/W = 1.51$. Moreover, we remark again that the pairing field influences on the characteristics of the spiral, optimizing its shape from block to canonical, to better host the MZM.

The behaviour of the edge-LDOS in the spiral phase needs special attention. As evident from the results presented in Supplementary Figure 6b, the value of the latter becomes finite for $\Delta_{SC}/W \gtrsim 0.25$ and vanishes for $\Delta_{SC}/W \sim 0.7$ (together with the already discussed $\boldsymbol{\kappa}_{L/2}$ and $\Delta_{T0}$). In order to explain the missing weight of edge LDOS for $\Delta_{SC}/W \lesssim 0.25$ let us investigate the frequency dependence of the hole $\langle\langle c_1 c_1^\dagger \rangle\rangle_\omega^{\mathrm{h}}$ and electron $\langle\langle c_1^\dagger c_1 \rangle\rangle_\omega^{\mathrm{e}}$ contributions to the edge-LDOS. As it is evident from the results in Supplementary Figure 7a, upon increasing $\Delta_{SC}$ the peaks in the electron- and hole-like spectral functions approach each other (Supplementary Figure 7b shows in more detail the positions of both maxima). Within the accessible frequency resolution, both peaks are easily distinguishable for $\Delta_{SC}/W \simeq 0.25$, while they merge into a single peak for $\Delta_{SC}/W > 0.3$.

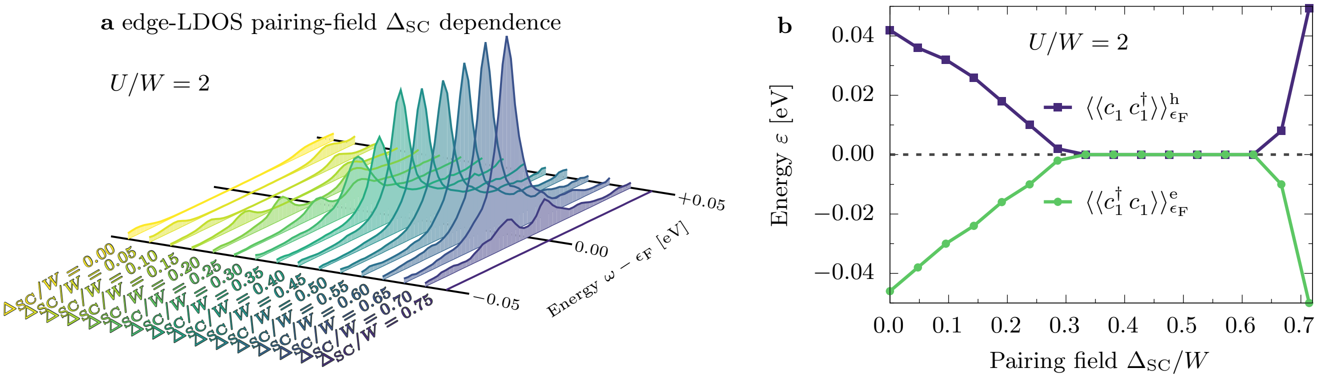


Supplementary Figure 7. **Pairing field dependence of the edge local density-of-states.** **a** Frequency $\omega$ dependence of edge ($\ell = 1$) local density-of-states (LDOS) as a function of the pairing field $\Delta_{SC}/W \simeq \{0.0\,, 0.05\,, \ldots, 0.7\}$, as calculated for $L = 36$, $\overline{n} = 0.5$, and $U/W = 2$. **b** Pairing field dependence of the maximum position [i.e., offset energy $\varepsilon$, see Eq. 14 of the main text] of the hole $\langle\langle c_1 c_1^\dagger \rangle\rangle_\omega^{\mathrm{h}}$ and electron $\langle\langle c_1^\dagger c_1 \rangle\rangle_\omega^{\mathrm{e}}$ contributions to edge-LDOS, see Eq. 8 of the main text (based on the data presented in panel **a**).

The behaviour described above is characteristic of systems hosting the MZM, i.e., despite the Majorana modes being

located at the opposite edges of the studied chain, they overlap in any finite-$L$ system [5, 6]. Due to this overlap, the peaks arise at a nonzero frequency $\omega = \pm\varepsilon$, see Eq. 14 of the main text. The clear splitting in the former case allows for a systematic finite-size study, shown in Supplementary Figure 8. In order to obtain well merged Majorana modes for $\Delta_{\mathrm{SC}}/W \simeq 0.25$, one needs to consider chains with at least $L \sim 60$ sites (see Supplementary Figure 8c), whereas systems half that size are sufficient for the case that is primarily studied in the present work, i.e., for $\Delta_{\mathrm{SC}}/W \simeq 0.5$ (see Supplementary Figure 8d). Furthermore, it is worth noting that if the chain is too short, then the remnants of the peaks become visible also in the middle of the system, as it is visible from results for $L = 24$ in Supplementary Figure 8a and Supplementary Figure 8c. On the other hand, a clear single peak at the system's edge always coincides with a well-developed gap in the bulk, as shown in Supplementary Figure 8b and Supplementary Figure 8d. All these results consistently support the scenario that the nonzero splitting $\varepsilon$ originates from the overlap of the edge modes. In Supplementary Figure 8e we explicitly show that $\varepsilon$ decays exponentially with $L$, as expected for systems hosting the MZM [5, 6].

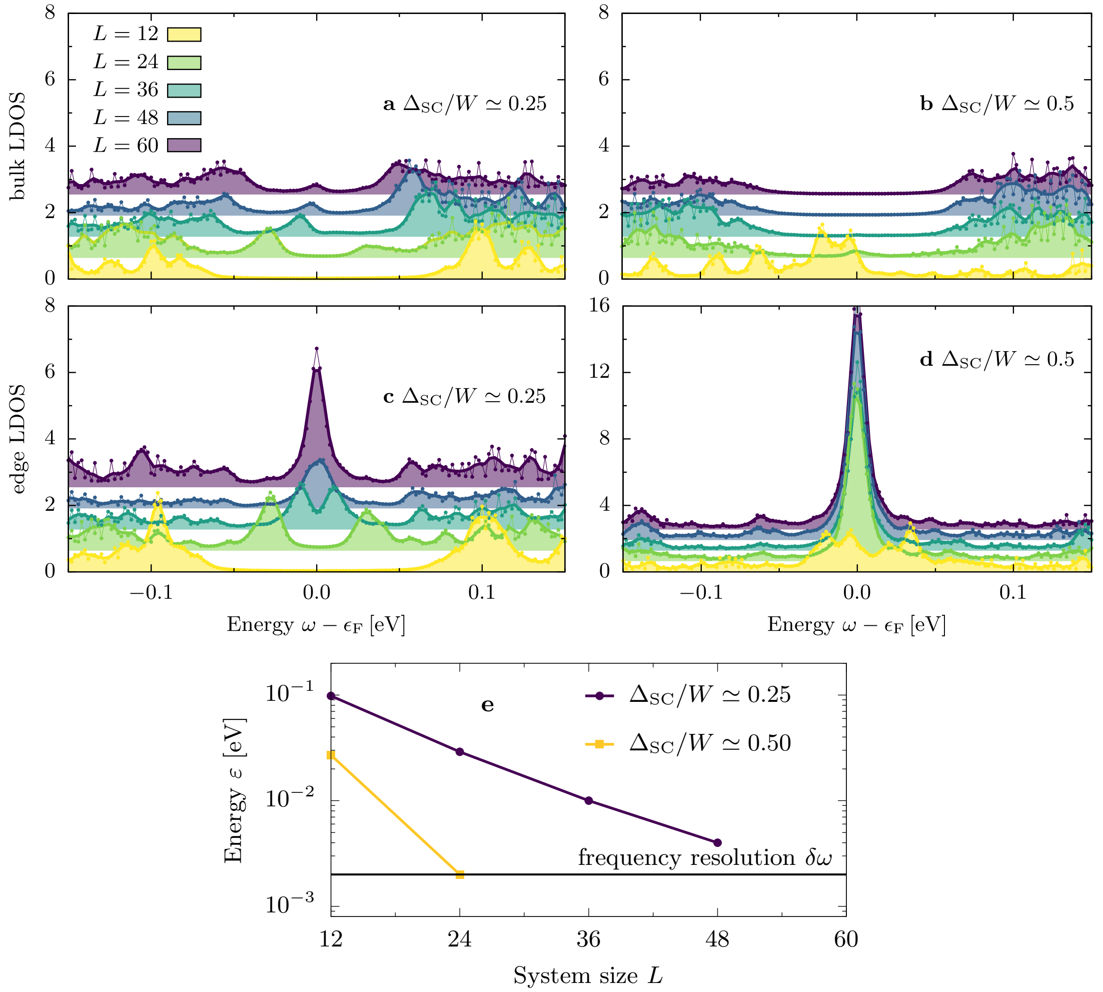


Supplementary Figure 8. **Finite-size analysis.** System lengths $L = \{12\,, 24\ldots, 60\}$ dependence of the local density-of-states **a-b** in the middle of the chain representing the bulk ($\ell = L/2$) and **c,d** at the edge ($\ell = 1$) of the system, as calculated for $U/W = 2$, **a,c** $\Delta_{\mathrm{SC}}/W \simeq 0.25$ and **b,d** $\Delta_{\mathrm{SC}}/W \simeq 0.5$. **e** System size $L$ dependence of the offset energy $\varepsilon$ [see Eq. 14 of the main text] for $\Delta_{\mathrm{SC}}/W \simeq \{0.25, 0.5\}$ (based on the data presented in panels **c** and **d**).

Finally, we discuss the robustness of our results to modifications in the localized orbital interaction strength $U_K$. Within our model, the latter manifests as a change in the spin exchange integral $K = 4t_1^2/U_K$. Our results, presented in Supplementary Figure 9a, indicate that when the system is in the trivial phase, $U/W = 1$ and $\Delta_{\mathrm{SC}}/W \simeq 0.5$, only a singlet SC amplitude is present for all considered values of $U_K/W \in [0.2, 3.0]$. For the topological phase at $U/W = 2$, the results (the presence of the triplet SC amplitude) do not depend on $U_K$ as long as the spiral nature of magnetism is not destroyed, i.e., for $U_K/W \gtrsim 0.8$. On the other hand, when the spin exchange integral $K \propto 1/U_K$ dominates as

an energy scale, the AFM ordering of the spins becomes energetically favorable [see Supplementary Figure 9b for the analysis of static structure factor $S(q)$] and, consequently, the system goes away from the topological phase. These results highlight the importance of the competing energy scales present in the multi-orbital OSMP system.

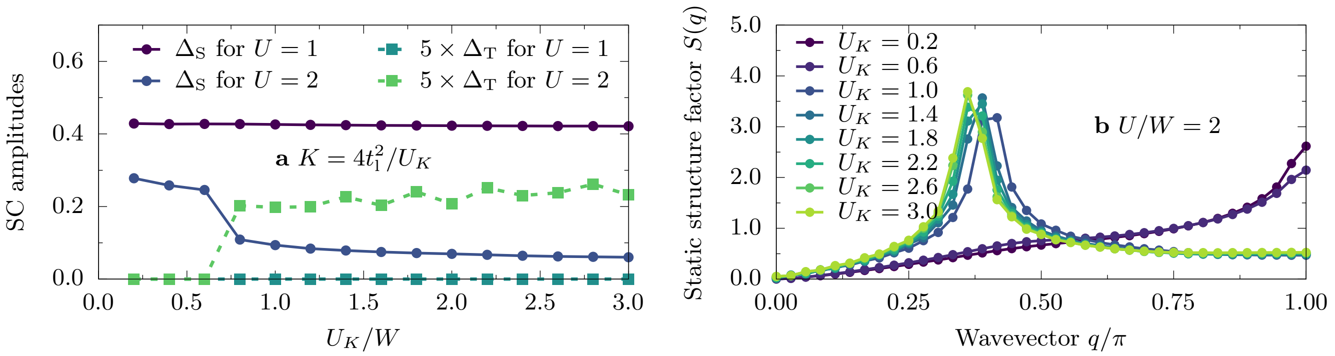


Supplementary Figure 9. **Spin exchange analysis.** **a** Dependence of the singlet $\Delta_S$ and triplet $\Delta_T$ SC amplitudes on the localized orbital interaction strength $U_K$, calculated for $L = 36$, $U/W = 1, 2$, $\overline{n} = 0.5$, and $\Delta_{SC}/W \simeq 0.5$. **b** Static spin structure factor $S(q)$ dependence on the localized orbital interaction strength $U_K$, calculated for $L = 36$, $U/W = 2$, $\overline{n} = 0.5$, and $\Delta_{SC}/W \simeq 0.5$.

## Supplementary Note 5. Entropy and dimer order

In this section, we demonstrate that the interaction-induced topological phase transition at $U_c$ may be identified via studying the entanglement entropy. Supplementary Figure 10a shows the dependence of the von Neumann entropy $S_{\mathrm{vN}}(\ell)$ on the subsystem size, $\ell \leq L$, in the vicinity of the transition, i.e., for $1.4 < U/W < 1.6$. Two characteristic behaviours emerge: for $U < U_{\mathrm{c}} \simeq 1.51W$ $S_{\mathrm{vN}}(\ell)$ displays an oscillatory behaviour, while for $U > U_{\mathrm{c}}$ the entropy increases abruptly and becomes a smooth function of $\ell$. This sudden change in the entropy behaviour signifies the interaction-induced topological phase transition and the appearance of MZM.

We also argue that the topological transition in the OSMP chain is accompanied by a rapid change of the dimer order $D_{\pi/2}$ (see Supplementary Figure 10b). In Supplementary Figures 10c and 10d we have shown the entanglement entropy, respectively for the trivial and nontrivial phases, where $S_{\mathrm{vN}}(\ell)$ displays clear oscillations in the former case. To explain the physical origin of such oscillations we have also plotted the static spin-spin correlation function $\langle \mathbf{T}_\ell \cdot \mathbf{T}_{\ell+1} \rangle$. We can observe that the maxima of $|\langle \mathbf{T}_\ell \cdot \mathbf{T}_{\ell+1} \rangle|$ and $S_{\mathrm{vN}}(\ell)$ coincide. Recall to calculate the entanglement entropy, we split the system into two subsystems cutting the bond between sites $\ell$ and $\ell+1$. Whenever a bond with a large spin-spin correlation is cut, also the entanglement entropy is large. Therefore, we expect that the oscillatory behaviour of $S_{\mathrm{vN}}(\ell)$ is a direct manifestation of the dimer order. Indeed, in the topological phase the spin dimerization persists only at the very edges of the system, as shown in Supplementary Figure 10d, so that the (bulk) dimer order vanishes presumably as $1/L$, see Supplementary Fig. 10b. Due to the absence of bulk dimer order, the entanglement entropy smoothly changes with $\ell$, as demonstrated also in Supplementary Figure 10a.

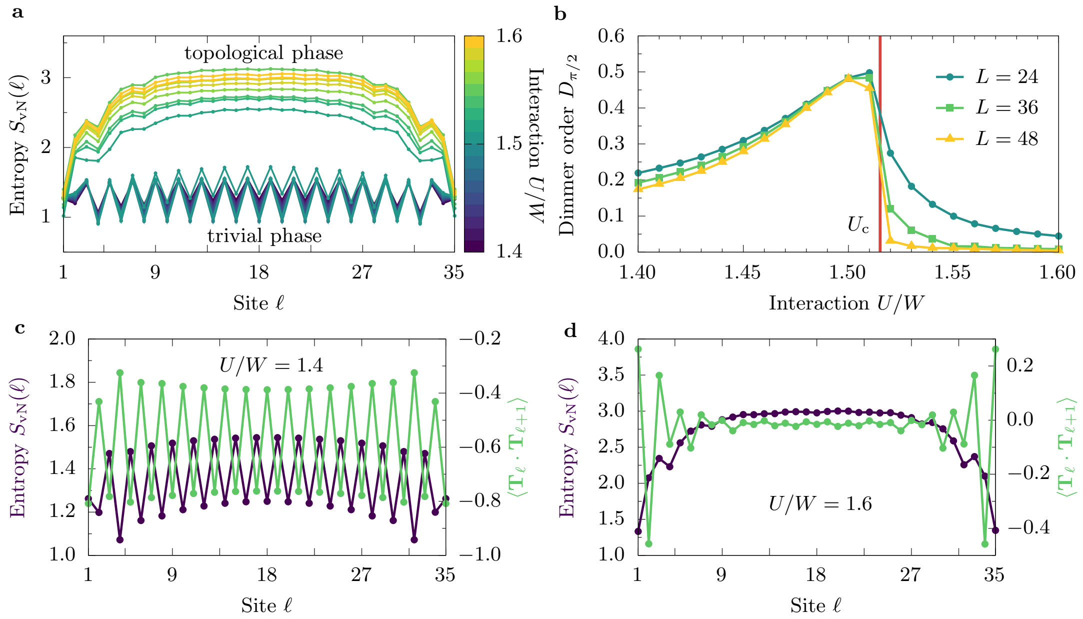


Supplementary Figure 10. **Entropy $S_{\mathrm{vN}}$ and dimer order $D_{\pi/2}$. a** Interaction $U \in \{1.41, 1.42, \ldots, 1.59, 1.60\}$ dependence of the von Neumann entanglement entropy $S_{\mathrm{vN}}(\ell)$ of the subsystem of size $\ell$. Calculated for $L = 36$, $\overline{n} = 0.5$, and $\Delta_{\mathrm{SC}}/W \simeq 0.5$. **b** Interaction dependence of the dimer order parameter $D_{\pi/2}$ as calculated for $L = 24, 36, 38$, $\overline{n} = 0.5$, and $\Delta_{\mathrm{SC}}/W \simeq 0.5$. **c-d** Site $\ell$ dependence of von Neumann entanglement entropy $S_{\mathrm{vN}}$ and local spin-spin correlation function $\langle \mathbf{T}_\ell \cdot \mathbf{T}_{\ell+1} \rangle$ (where $\mathbf{T}_\ell = \mathbf{S}_\ell + \boldsymbol{s}_\ell$ is the total on-site spin) **c** below ($U/W = 1.4$) and **d** above ($U/W = 1.6$) the topological phase transition. Results calculated for $L = 36$, $\Delta_{\mathrm{SC}}/W \simeq 0.5$, and $\overline{n} = 0.5$.

Furthermore, the vanishing of the dimer order can also be observed in the behaviour of the chirality correlation function $\langle \boldsymbol{\kappa}_{L/2} \cdot \boldsymbol{\kappa}_\ell \rangle$ shown in Supplementary Figure 11. As expected in the $\Delta_{\mathrm{SC}} \to 0$ limit (for which $D_{\pi/2} \neq 0$), the $\langle \boldsymbol{\kappa}_{L/2} \cdot \boldsymbol{\kappa}_\ell \rangle$ correlation displays a clear zig-zag–like pattern, reflecting the $\pi/2$-block nature of the spiral (for details see Ref. [3]). It is evident from the presented results that the decay length of the $\boldsymbol{\kappa}$-correlation is not affected by the pairing field strength. On the other hand, the spatial details of $\langle \boldsymbol{\kappa}_{L/2} \cdot \boldsymbol{\kappa}_\ell \rangle$ change from a zig-zag to a smooth function of distance. The latter is consistent with the $D_{\pi/2} \to 0$ result in this region.

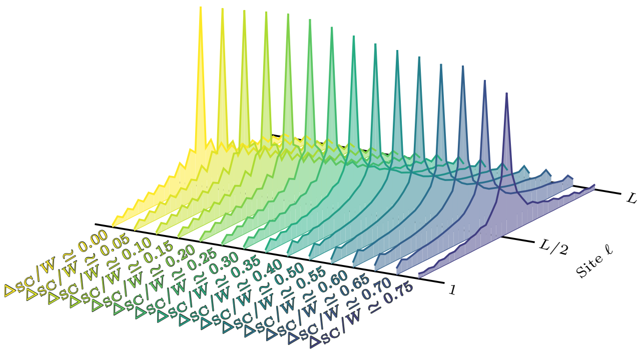


Supplementary Figure 11. **Pairing field dependence of the spiral order.** Site dependence of the chirality correlation function $\langle \boldsymbol{\kappa}_{L/2} \cdot \boldsymbol{\kappa}_{\ell} \rangle$ as calculated for various strengths of the SC pairing fields $\Delta_{\rm SC}/W \simeq (0.00, 0.05, \ldots, 0.75)$ ($\overline{n} = 0.5$, $U/W = 2$, $L = 36$).

[1] Herbrych, J. *et al.* Spin dynamics of the block orbital-selective Mott phase. *Nat. Commun.* **9**, *3736 (2018)*.

[2] Herbrych, J. *et al.* Novel Magnetic Block States in Low-Dimensional Iron-Based Superconductors. *Phys. Rev. Lett.* **123**, *027203 (2019)*.

[3] Herbrych, J. *et al.* Block-Spiral Magnetism: An Exotic Type of Frustrated Order. *Proc. Natl Acad. Sci. USA* **117**, *16226 (2020)*.

[4] Lei, H., Ryu, H., Frenkel, A. I. & Petrovic, C. Anisotropy in $BaFe_2Se_3$ single crystals with double chains of FeSe tetrahedra. *Phys. Rev. B* **84**, *214511 (2011)*.

[5] Stanescu, T. D. , Lutchyn, R. M. & Das Sarma, S. Dimensional crossover in spin-orbit-coupled semiconductor nanowires with induced superconducting pairing. Phys. Rev. B **87**, 094518 (2013).

[6] Rainis, D., Trifunovic, L., Klinovaja, J. & Loss, D. Towards a realistic transport modeling in a superconducting nanowire with Majorana fermions. Phys. Rev. B **87**, 024515 (2013).
